# Strong interactions between carbones and halogen atomic centers

**DOI:** 10.1039/d6sc02210c

**Published:** 2026-06-19

**Authors:** Shunhua Li, Hangyu Zhou, Qingzhong Li, Steve Scheiner

**Affiliations:** a The Laboratory of Theoretical and Computational Chemistry, School of Chemistry and Chemical Engineering, Yantai University Yantai 264005 P. R. China lqz@ytu.edu.cn; b Department of Chemistry and Biochemistry, Utah State University Logan UT 84322-0300 USA steve.scheiner@usu.edu

## Abstract

Carbone compounds are characterized by a zero oxidation state on divalent C, which contains two lone pairs, coupled with dative bonding to its two substituents. The ability of this carbone center to act as an electron donor is examined by pairing it with 29 different halogen-containing Lewis acids. DFT calculations show the binding to be quite strong, eclipsing that of a NH_3_ Lewis base, despite the similarity of their electrostatic potentials. Binding energies span a wide range from nearly zero up to more than 40 kcal mol^−1^; interaction energies are even larger. The more weakly bound dyads, with binding energies below 20 kcal mol^−1^, have all the characteristics of conventional halogen bonds, including an increase in binding energy in the usual Cl < Br < I sequence. For the more powerful Lewis acids, there is a progressively larger degree of displacement of the halogen atom from the Lewis acid to the carbone center, some essentially fully transferred. The dependence of the energetics on the halogen atom reverses for these complexes: Cl > Br > I.

## Abstract 2

The divalent C(0) atom with zero oxidation state is a powerful electron donor, capable of forming very strong halogen bonds, or even ripping the halogen off of its partner.

## Graphical abstract

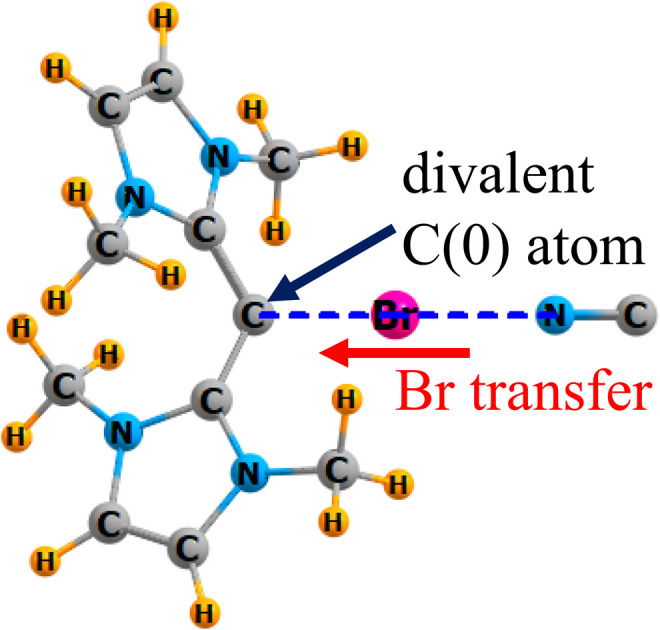

## Introduction

The halogen bond (XB) has been widely recognized in recent years. Its origin lies first in the formation of a region of positive electrostatic potential, known as the σ-hole,^1^ along the projection of the R–X covalent bond with halogen atom X = Cl, Br, or I. This σ-hole acts as a Lewis acid site, which electrostatically attracts a Lewis base which imparts a strong directionality to the XB.^2–4^ Consequently, it has become a precise design tool in fields such as protein recognition,^5^ targeted drug design,^6^ and the construction of functionalized organic framework materials.^7^

Implementation of the XB has promoted breakthrough progress in the creation of functional materials. Chen *et al.*^8^ skilfully utilized XB interactions to successfully construct a halogen-bonded organic framework material, I^+^(C_6_H_4_NO_2_)^−^(INA). The INA in this material exhibits excellent birefringence performance. This discovery opens a novel halogen-bond-driven strategy for the design and synthesis of a new generation of high-performance optical birefringent crystals. Furthermore, XB also demonstrates considerable potential in the field of energy materials. Yang *et al.*^9^ constructed a halogen-bonded azo-based cathode material, 4,4′-azopyridine-iodide (AZPY-I). This material maintains exceptional cycling stability even under ultra-high current densities, highlighting the unique advantages of XBs in stabilizing electrode material structures and enhancing fast charge/discharge performance. It is now clear that the value of halogen bonding extends far beyond technological breakthroughs in a single field. Its deeper scientific significance lies in providing a programmable, modular paradigm for molecular assembly. By precisely tuning the electronic properties of halogen donors and the spatial geometry of acceptors, researchers can achieve rational design of materials, from their mesoscale structures to macroscopic properties.

Carbon, a non-metallic element abundant in nature, possesses a unique electronic structure with four valence electrons, enabling it to adopt various hybridizations and oxidation states. This versatility enables carbon to form complex covalent networks and exhibit diverse electronic behaviours. It can act as a Lewis acid to accept electron pairs or, under specific conditions, as a Lewis base to donate lone pairs of electrons. For quite some time, carbon's potential as an electron donor was largely underappreciated, due in part to its moderate electronegativity and low polarizability. However, with the rapid development of modern experimental techniques and theoretical computational methods, the directional interactions involving carbon as an electron donor have been gaining growing attention. Echeverría^10^ observed that during the interaction between alkyl groups and the Lewis acid ER_3_ (E = B, Al, Ga, In, Tl), the measured distances are shorter than the sum of the van der Waals radii but significantly longer than the sum of the covalent radii. They deduced that sp^3^-hybridized carbon atoms can act as electron donors, laying the groundwork for the application of carbon atoms as novel structural units in supramolecular design. In 2021, Scheiner^11^ provided a systematic summary and classification of carbon's role as an electron donor. The review highlighted that various carbon-containing entities—including CN^−^, a derivative of an alkane from which a proton has been removed, metal-substituted methane (*e.g.*, CH_3_Li), carbenes, and π-systems—can all serve as effective electron donors. This broadens the scope of carbon's utility as a Lewis base and paves the way for further research. Recent studies have expanded into applications in fields such as environmental catalysis,^12–14^ energy conversion^15,16^ and optoelectronic materials.^17^ Carbon atoms, particularly in graphene, have shown central roles in activating permanganates for pollutant degradation and in stabilizing metal centres to enhance emission properties in organic light-emitting diodes (OLEDs).^18^

The manifestation of carbon as an electron donor extends far beyond these examples. The classic divalent carbon compound—carbene (:CR_2_)—is a quintessential representative, serving as a carbon-based electron donor.^19–21^ Its central carbon atom possesses a σ-type lone pair and a formal oxidation state of +2. Major progress in divalent carbon(ii) chemistry was achieved in 1991 when Arduengo *et al.*^22^ introduced imidazole-2-ylidene as a synthetically useful, stable molecule. This built upon the earlier isolation of a stable carbene by Bertrand *et al.*^23–26^ in 1985. Together, these works significantly expanded the scope of divalent carbon(ii) chemistry.^27,28^

Early work by Kaufhold and Hahn^29^ proposed a series of compounds in which a divalent carbon can be best described by two resonance structures. The formal oxidation state of the central C is −2 when it is surrounded by two single bonds and two lone pairs, as compared to the single lone pair within a carbene. An alternate structure surrounds this C by two double bonds, and no lone pairs, leaving it with an oxidation state of 0. Frenking *et al.*, elucidated the unique nature of this type of species through a detailed study of model compounds like carbodiphosphorane C(PPh_3_)_2_.^30–32^ Their calculations favored an electronic structure with oxidation state of zero. The two C lone pairs were of two types, one σ and the other π, a fundamental distinction from the single σ-type lone pair in a carbene. This type of molecule is now more commonly referred to as a carbone.^33–35^

C(NHC_Me_)_2_ is a two-coordinate carbodicarbene^36^ (a subclass of carbone) with a bent geometry that features a central carbon atom coordinated by two methyl-substituted *N*-heterocyclic carbene ligands. This compound has a shallow bending potential and exhibits good structural flexibility, allowing easy rotation of the ligands and variation in bond angles. Despite formally having a C=C=C double-bond electronic structure, its frontier orbitals still show some indication of lone-pair character on the central carbon.^29,30^ Klein *et al.*^37^ thoroughly investigated the Lewis basicity of C(NHC_Me_)_2_. At the BP86/SVP level, they found that the dissociation energy of the monocoordinated complex C(NHC_Me_)_2_-BH_3_ is as high as 42.1 kcal mol^−1^. Even removing one BH_3_ from the dicoordinate complex BH_3_-C(NHC_Me_)_2_-BH_3_ requires a dissociation energy of 29.0 kcal mol^−1^. This result is supported by the work of Esterhuysen *et al.*,^38^ who confirmed the very strong basicity of C(NHC_Me_)_2_ as a Lewis base. They specifically pointed out that its divalent carbone nature can be distinguished from alkenes by its coordination mode with AuCl: it can bind one or two AuCl groups in an *η*^1^-fashion, whereas alkenes bind in an *η*^2^-fashion. These high dissociation energies indicate that C(NHC_Me_)_2_ is a strong Lewis base. More importantly, they reveal its unique dual-centre electron-donating ability—it can simultaneously and stably coordinate two Lewis acid molecules while maintaining high bond strength. This provides an important theoretical foundation for its potential applications in advanced fields such as bifunctional catalysis and cooperative small-molecule activation.^39–41^

There are several fundamental questions that arise concerning this molecule, and other carbone molecules in general. In the first place, how strong a halogen bond can it form with an incoming Lewis acid? What is the nature of this bonding, and how does it relate to the unique electronic structure of carbone molecules? What sort of perturbations does the carbone halogen bonding introduce into the Lewis acid molecule? How does this electronic structure elevate its ability to act as an electron donor, and how does this carbone center compare with other more electronegative atoms like N or O? These questions are probed here by high-level DFT calculations that pair C(NHC_Me_)_2_ with 29 different halogen-containing molecules that span a wide range of electrophilicity. One of the more interesting results to emanate from these calculations is the ability of this carbodicarbene to pry the halogen atom off the Lewis acid, causing it to transfer across to the C atom to varying degrees.

## Theoretical methods

All calculations were performed at the BP86/def2-TZVP^42–44^ level of theory using the Gaussian 09 program.^45^ For the heavier iodine(i) atom, the def2-TZVP basis set includes an effective inner shell pseudopotential. Geometries of all structures were fully optimized, followed by frequency calculations to confirm that they are true local minima on the potential energy surface (no imaginary frequencies). Natural charges *q*(X) of the monomers, corresponding to the charge on the halogen atom X, as well as the charge transfer and associated inter-orbital interactions within the complexes, were evaluated by natural bond orbital (NBO) analysis.^46^ The dissociation energies *E*_dissoc_ for the removal of X^+^ were calculated as the energy difference between the acid and the fragments (X^+^ and the remaining anion). For the open-shell singlet state of X^+^, the unrestricted formalism (UBP86) was employed with the guess = mix keywords to ensure a stable wavefunction. The interaction energy was calculated as the difference between the energy of the complex and the sum of the energies of the monomers, with the monomers fixed in their geometry from the complex. This value was corrected for basis set superposition error (BSSE) using the counterpoise procedure proposed by Boys and Bernardi.^47^ The binding energy was defined analogously, with the key difference being that the monomers were held at their fully optimized, isolated geometries.

The molecular electrostatic potential (MEP) of each monomer was analysed using the Multiwfn^48,49^ program. Based on the Quantum Theory of Atoms in Molecules (QTAIM),^50–52^ the topological properties at relevant bond critical points (BCPs) were analysed, including the electron density (*ρ*), its Laplacian (∇^2^*ρ*), and total energy density (*H*). Noncovalent interaction (NCI)^53–55^ plots were generated using Multiwfn and visualized with VMD.^56^

The energy decomposition analysis (EDA)^57^ method combined with the Extended Transition State-Natural Orbitals for Chemical Valence (ETS-NOCV) theory^57–60^ was employed to provide an in-depth analysis of the nature of the XB interaction. The EDA method was implemented in the Amsterdam Density Functional (ADF 2013) program package^61,62^ and was used to analyze the halogen bond between a carbone center and X (X = Br, Cl, I). Single-point energy calculations were performed using the BP86-D^63^/TZ2P method in the ADF 2013 software, based on geometries optimized with the BP86/def2-TZVP method in the Gaussian software. This EDA method decomposed the total interaction energy (Δ*E*_int_) into four physically meaningful components:^57^ electrostatic interaction energy (Δ*E*_elstat_), which represents the quasi-classical electrostatic interaction between two fragments, primarily arising from the attractive interaction between electrons on one fragment and the nucleus of the other; Pauli repulsion energy (Δ*E*_Pauli_), which originates from the repulsive interaction between occupied molecular orbitals of the two fragments, reflecting the energy increase due to the Pauli exclusion principle; orbital interaction energy (Δ*E*_orb_), which describes the charge transfer and polarization effects between occupied molecular orbitals of one fragment and unoccupied molecular orbitals of the other, and is typically closely associated with covalent bond formation; and dispersion interaction energy (Δ*E*_disp_), which corresponds to the long-range dispersion effect between two fragments, namely the instantaneous dipole-induced dipole interaction. The Δ*E*_orb_ term can be further partitioned into individual energy contributions corresponding to specific orbital interactions, thereby allowing the differentiation of σ, π, and δ bond interactions. Energy decomposition analysis was also conducted based on the Generalized Kohn–Sham Energy Decomposition Analysis (GKS-EDA)^64^ theory, implemented *via* the XEDA^65^ software, with input files generated by the Mokit program.

## Results

### Monomers

**Fig. 1:**
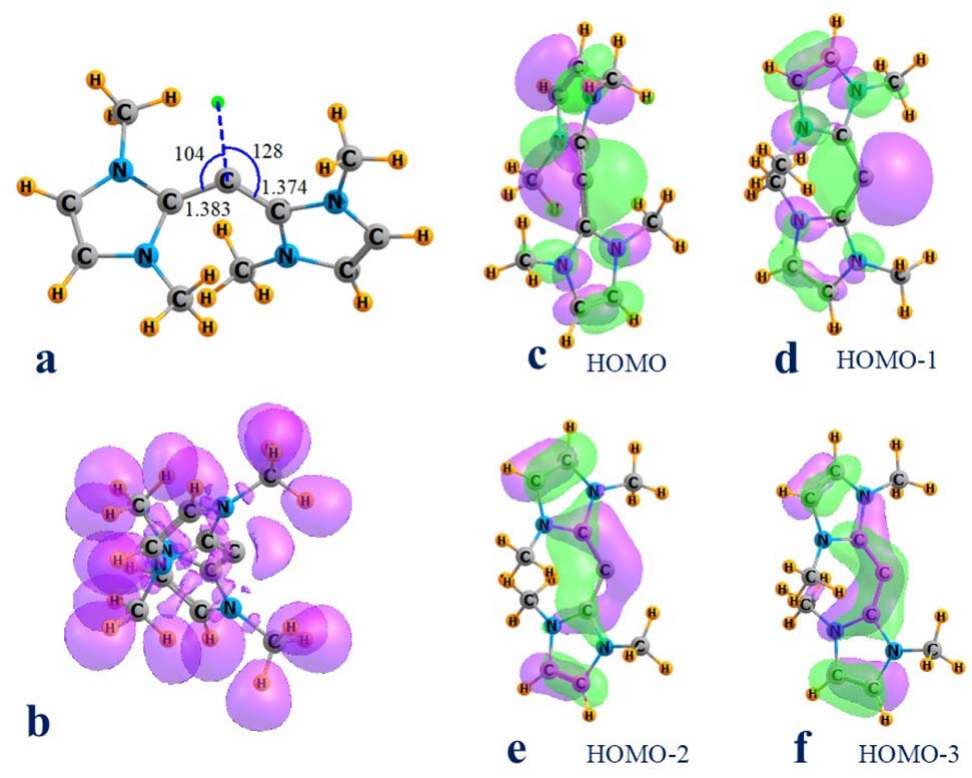
(a) Optimized geometry of CLB. Small green ball indicates position of *V*_s,min_. Bond lengths are in Å, angles in degs. (b) ELF diagram. Various orbitals are presented in (c–f).

**Fig. 2:**
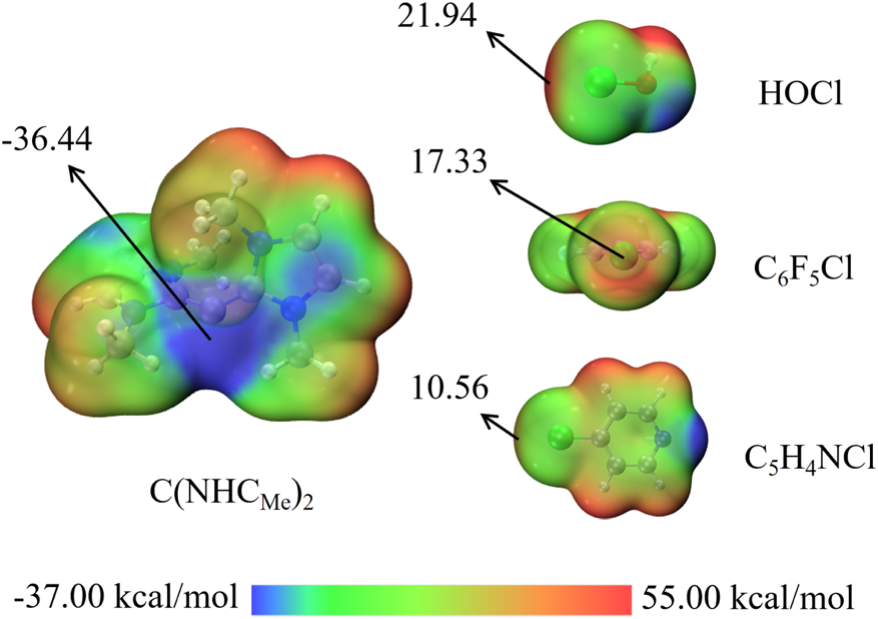
Molecular electrostatic potential (MEP) maps of several monomers, highlighting electron-rich (blue) and electron-deficient (red) regions. The color-coded scale ranges from −37 to +55 kcal mol^−1^, specifically: blue (−37 to −22), green (−22 to +8), and red (8 to 55). Numbers indicate values of *V*_s,min_ and *V*_s,max_, in kcal mol^−1^.

Fig. 1a illustrates the optimized geometry of the carbon-centered Lewis base C(NHC_Me_)_2_, designated below as CLB. The two imidazole rings are skewed, with the two *θ*(CCCN) dihedral angles equal to 30° and −50°, and the two C–C bond lengths to the central carbon differ by 0.009 Å. The molecular electrostatic potential (MEP) surrounding CLB is displayed on the left side of Fig. 2, highlighted by a strongly negative blue region near the central C. This lump can be quantified as a minimum in the MEP on a 0.001 au isodensity surface, *V*_s,min_ equal to −36.44 kcal mol^−1^, represented by the small green ball in Fig. 1a. This minimum is located in the C–C–C plane but is not quite symmetrical, displaced toward the ring on the left by a few degrees. The ELF diagram of the molecule in Fig. 1b shows a region of electron density centered along the C–C–C bisector, that spans into the π-region above and below the C–C–C plane to some extent.

The HOMO and HOMO−1 of CLB are contained in Fig. 1c and d, each resembling a lone pair on the central C atom. The HOMO is of π-character with lobes of opposite sign on either side of the C, while the HOMO−1 orbital is σ, with one lobe pointing toward the minimum in its MEP. The bonding around this central C is supplemented by the two orbitals immediately below them in energy. The HOMO−2 in Fig. 1e is a sort of 3-center CCC σ-bonding orbital, while its π-equivalent occurs in the HOMO−3 of Fig. 1f. NBO description of the bonding within this molecule fits the bent C=C=C resonance picture, with a pair of classical localized double bonds, one σ and one π, and with no lone pairs.

There are 29 different halogen-containing Lewis acids XR that were paired with CLB. For asymmetric dihalogen molecules (XF, XCl, X = Br, Cl, I), the σ-hole donor is the halogen atom with the lower electronegativity (*i.e.*, the one written first in the molecular formula). The MEPs surrounding three of these acids are displayed on the right side of Fig. 2 for illustrative purposes. The principal feature of interest here is the red positive region near the X atom, referred to as a σ-hole. *V*_s,max_ is defined as the maximum in the MEP on the 0.001 au isodensity surface, and is collected in the first column of Table 1 for all 29 of these Lewis acids. *V*_s,max_ increases with the size, polarizability and electropositivity of the halogen atom (from Cl to I). The deepest σ-holes are associated with F, CN, and NC substituents, all strong electron-withdrawing agents. The electron-donating NH_2_ and C_5_H_4_N groups yield the smallest values of *V*_s,max_, although these quantities still exceed 9 kcal mol^−1^. Another aspect of these acids is the natural charge associated with the halogen atom, listed as *q*(X) in Table 1. The last column of Table 1 contains the dissociation energy required to pull a X^+^ off the acid; the relevance of this quantity will be apparent below.

**Table 1:** Maximum of the MEP (*V*_s,max_, kcal mol^−1^) on the 0.001 au isodensity surface near the halogen X atom, natural charge of X, *q*(X) (*e*), and *E* required to remove X^+^ (kcal mol^−1^)

	*V*_s,max_	*q*(X)	*E*_dissoc_		*V*_s,max_	*q*(X)	*E*_dissoc_
Cl_2_	25.63	0.000	297.9	ClCN	35.94	0.174	334.0
Br_2_	29.73	0.000	262.6	BrCN	41.06	0.230	295.1
I_2_	32.25	0.000	227.6	ICN	48.03	0.313	254.0
ClF	40.19	0.312	312.9	ClNH_2_	9.04	0.052	374.4
BrF	47.99	0.387	286.3	BrNH_2_	16.89	0.111	340.4
IF	54.82	0.487	259.4	INH_2_	25.16	0.199	302.8
BrCl	34.97	0.094	267.2	ClCF_3_	18.37	−0.028	386.5
ICl	44.03	0.215	234.5	BrCF_3_	22.14	−0.007	346.5
ClNC	38.70	0.293	291.6	ICF_3_	29.26	0.047	300.4
BrNC	45.69	0.352	258.8	ClC_6_F_5_	17.33	0.090	343.4
INC	53.24	0.444	225.7	BrC_6_F_5_	23.14	0.143	304.3
ClOH	21.94	0.172	346.1	IC_6_F_5_	31.13	0.232	261.4
BrOH	30.35	0.246	315.7	ClC_5_H_4_N	10.56	0.034	383.9
IOH	38.37	0.347	283.1	BrC_5_H_4_N	15.01	0.080	344.2
				IC_5_H_4_N	21.90	0.156	299.1

### Geometric structures

**Fig. 3:**
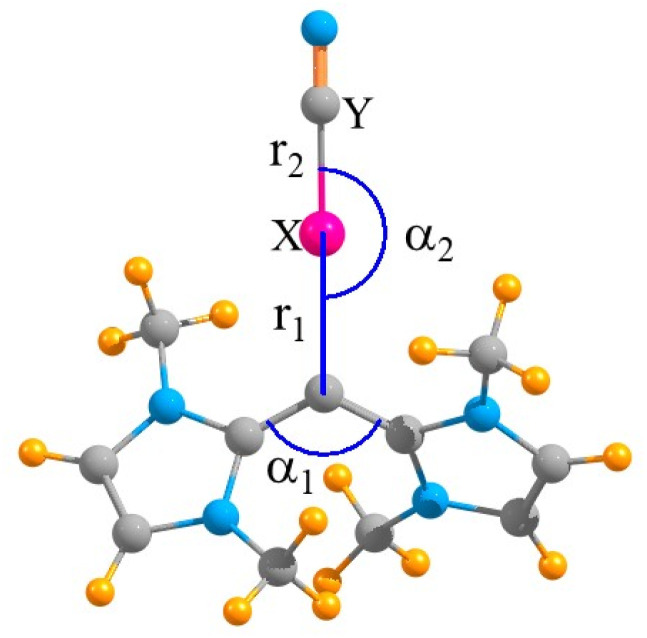
Definition of geometric parameters of complex.

When placed near the central C atom of CLB, each of the Lewis acids forms a complex of the form depicted in Fig. 3. The α_2_ angle describes the linearity of the C⋯XY halogen bond arrangement. The values contained in the first column of Table 2 are close to 180° with one main exception to be explained below. The formation of the XB induces a small contraction of the C–C–C angle α_1_, on the order of 10° or less. The *r*_1_ distance refers to the length of the XB, and the intramolecular XY bond length within the Lewis acid unit is represented by *r*_2_.

**Table 2:** Distances (*r*, Å), angles (*α*, degs), and their changes caused by complexation with CLB

	*α*_2_	Δ*α*_1_	*r*_1_	*r*_2_	Δ*r*_1_^a^	Δ*r*_2_	*t* = Δ*r*_2_/Δ*r*_1_
Cl_2_	180.0	−6.2	1.880	2.591	0.111	0.570	5.16
Br_2_	180.0	−7.2	2.110	2.749	0.174	0.430	2.47
I_2_	180.0	−8.7	2.360	3.022	0.223	0.330	1.48
ClF	180.0	−7.2	1.900	2.044	0.131	0.380	2.91
BrF	180.0	−8.5	2.120	2.067	0.184	0.280	1.52
IF	180.0	−10.3	2.340	2.130	0.203	0.190	0.94
BrCl	180.0	−7.5	2.107	2.580	0.171	0.412	2.41
ICl	180.0	−9.5	2.339	2.645	0.202	0.297	1.47
ClNC	153.9	−7.0	1.775	2.838	0.006	1.205	—
BrNC	180.0	−8.2	2.062	2.329	0.126	0.530	4.20
INC	180.0	−10.2	2.317	2.331	0.180	0.343	1.91
ClOH	179.0	−5.8	2.040	2.049	0.271	0.330	1.22
BrOH	178.7	−7.5	2.250	2.095	0.314	0.240	0.76
IOH	178.4	−9.4	2.450	2.182	0.313	0.170	0.54
ClCN	175.6	−4.8	2.824	1.672	1.055	0.038	0.04
BrCN	180.0	−7.6	2.454	1.990	0.518	0.194	0.37
ICN	180.0	−9.5	2.510	2.223	0.373	0.225	0.60
ClNH_2_	175.7	−4.9	2.481	1.933	0.712	0.159	0.22
BrNH_2_	176.1	−6.5	2.499	2.095	0.563	0.166	0.29
INH_2_	176.1	−8.4	2.623	2.239	0.486	0.134	0.28
ClCF_3_	175.4	−3.3	2.980	1.792	1.211	0.010	0.01
BrCF_3_	176.1	−5.8	2.770	2.021	0.834	0.060	0.07
ICF_3_	179.9	−8.3	2.660	2.289	0.523	0.110	0.21
ClC_6_F_5_	175.6	−3.4	2.970	1.750	1.201	0.030	0.02
BrC_6_F_5_	180.0	−6.6	2.550	2.029	0.614	0.140	0.23
IC_6_F_5_	180.0	−8.7	2.560	2.275	0.423	0.180	0.43
ClC_5_H_4_N	175.4	−1.8	3.363	1.745	1.594	0.002	0.00
BrC_5_H_4_N	175.4	−3.6	3.090	1.931	1.154	0.023	0.02
IC_5_H_4_N	176.0	−6.5	2.945	2.168	0.808	0.055	0.07

a relative to CLB-X^+^ cation.

Of some interest is the stretch of the internal covalent bond caused by the formation of the halogen-bonded dyad, relative to the bond length within each isolated monomer. This quantity is listed in Table 2 as Δ*r*_2_, and is highly variable, ranging between 0.002 and 0.570 Å. (The ClNC system is exceptional as described in greater detail below.) Also of interest is the stretch of the central X from the CLB. This quantity is defined as Δ*r*_1_, the difference between *r*_1_ within the complex and the C–X bond length for the CLB-X^+^ cation, *i.e.* where the X lies fully on the base in the absence of the remainder of the Lewis acid molecule. Δ*r*_1_ is rather large for most dyads, not surprising in view of the fact that X is normally an integral part of the acid molecule, covalently attached to Y. (Of course, a straight comparison between *r*_1_ and *r*_2_ themselves would be complicated by the differing sizes of C and the Y center. It is for this reason that it is Δ*r*_1_ and Δ*r*_2_ that are the focus here.)

By comparing these two bond stretch parameters, in the form of the Δ*r*_2_/Δ*r*_1_ ratio, defined here as *t*, one can formulate a quantitative measure of the degree of halogen transfer, from Y to C. A very small ratio would signal that the central X has moved very little from its position close to Y, whereas this ratio would grow continuously larger as X moves along the Y⋯C axis toward C. A value of 1 might be considered as half transfer as the halogen atom would be equally removed from both Y and the C of CLB. This quantity is fairly large near the top of Table 2 and drops down quickly toward the bottom and even vanishes for ClC_5_H_4_N. The absence of some entries for ClNC is due to the fact that the geometry optimization of its dyad with CLB leads to a full transfer of the Cl, leading to a 1.2 Å increase of the N⋯Cl distance, followed by a displacement of the remaining CN subunit away from the Cl and toward a methyl group. It is for this reason that α_2_ is equal to 154° for this dyad.

There are some clear patterns evident in *t*, which seems to divide the systems into two subsets. For those above the ClCN row in Table 2, *t* is fairly large, at least 0.5. It is largest for the X_2_ dihalogens, followed by XNC and then XF, and finally XOH. For each particular Lewis acid type, the transfer parameter follows the order Cl > Br > I. In other words the lighter X atoms are more prone to transfer. The systems in the lower portion of Table 2 below the ClCN row follow an opposite pattern: Cl < Br < I. This ratio is generally smaller than for those in the upper section of the table, all below 0.6, suggesting a considerably lesser degree of halogen transfer, and one in which the heavier X atoms are more mobile than Cl.

### Energetics of dyads

The interaction energies of the various complexes are compiled in the first column of Table 3. These quantities are quite large, all over 30 kcal mol^−1^, and some over 70 kcal mol^−1^. The outlier for ClNC is again due to the full transfer of the Cl across to the CLB, so is not a good indicator of the true interaction. The binding energies in the second column of data take into account the deformation energies (DE) needed for the monomers to adjust their geometries from their fully optimized structure to that within the complex. These deformation energies are particularly large for those near the top of Table 3 which involve a large stretch of the X–Y distance. For the upper set of systems, *E*_int_ becomes smaller in magnitude in the Cl > Br > I sequence, as does *E*_b_ (except for the XOH acids). This trend reverses for the systems below the ClCN row of Table 3, with the I > Br > Cl consistent with the normal pattern of XBs. In brief the upper set of complexes all have binding energies above the 20 kcal mol^−1^ mark, while those of the lower set fall below this threshold, some even approaching zero.

**Table 3:** Interaction energy (*E*_int_), binding energy (*E*_b_), and deformation energy (DE) for complexes with CLB, all in kcal mol^−1^, and change in X–Y stretching frequency (Δ*ν*, cm^−1^) caused by complexation

	*E*_int_	*E*_b_	DE	Δ*ν*
Cl_2_	−73.78	−38.01	35.77	−255.00
Br_2_	−57.24	−35.52	21.72	−134.30
I_2_	−43.90	−29.97	13.93	−57.70
ClF	−73.54	−44.81	28.73	−392.50
BrF	−60.42	−42.16	18.26	−283.40
IF	−50.39	−38.25	12.14	−197.20
BrCl	−59.39	−36.80	22.59	−216.80
ICl	−50.09	−35.01	15.08	−150.30
ClNC	−115.75	−37.37	78.38	—
BrNC	−68.58	−35.69	32.89	−325.80
INC	−54.32	−34.94	19.38	−225.40
ClOH	−40.24	−20.39	19.85	−713.40
BrOH	−35.48	−22.73	12.75	−614.60
IOH	−32.88	−23.68	9.20	−563.10
ClCN	−6.56	−5.13	1.43	−139.80
BrCN	−22.25	−10.84	11.41	−290.20
ICN	−31.04	−18.16	12.88	−213.10
ClNH_2_	−9.37	−3.41	5.96	−257.90
BrNH_2_	−15.07	−8.24	6.83	−187.40
INH_2_	−18.41	−11.94	6.47	−133.10
ClCF_3_	−2.82	−2.06	0.76	−48.10
BrCF_3_	−8.46	−5.46	3.00	−77.70
ICF_3_	−18.47	−11.26	7.21	−73.10
ClC_6_F_5_	−2.71	−1.88	0.83	−17.20
BrC_6_F_5_	−13.68	−6.25	7.43	−33.10
IC_6_F_5_	−22.94	−12.92	10.02	−35.50
ClC_5_H_4_N	−0.17	−0.02	0.15	−9.30
BrC_5_H_4_N	−2.45	−1.73	0.72	−29.20
IC_5_H_4_N	−7.76	−5.43	2.33	−36.60

**Fig. 4:**
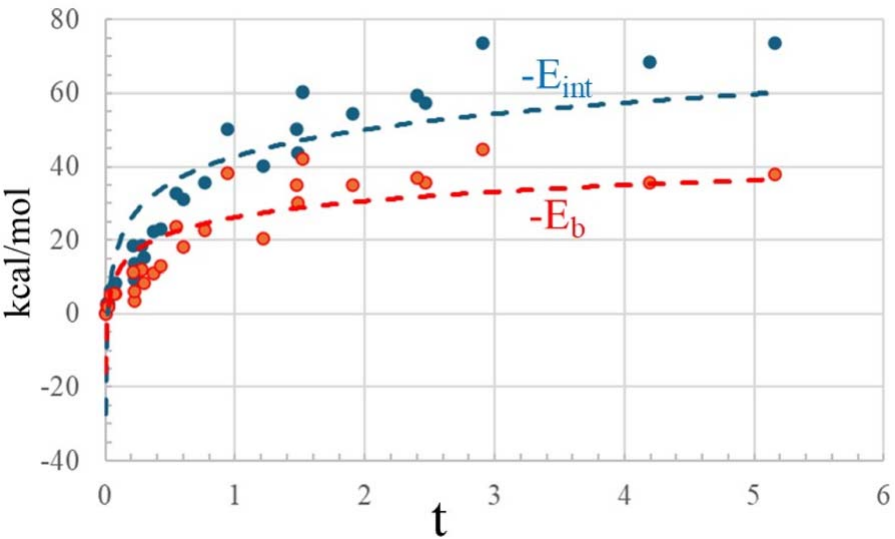
Relationship between energetics of complexation and halogen transfer parameter t.

There is a clear relationship between the energetics in Table 3 and the degree of halogen transfer in each complex. Both the interaction and binding energies are plotted against the transfer parameter in Fig. 4. As explained above, *E*_int_ is larger in magnitude than *E*_b_, due to the deformation which the XY unit must undergo. But both quantities show a similar pattern of growing quickly as the X transfers across to CLB. This growth continues well past *t* = 1 as the half transfer marker, eventually reaching a plateau as *t* exceeds 2, and the transfer nears completion. Given the strong relationship between the energetics of the binding and the degree of halogen transfer, it was thought that there might perhaps also be some correlation with the charge that this X centre bears within the Lewis acid molecule, or the degree of difficulty of removing a X^+^ from that unit. The former quantity is listed in Table 1 as the natural charge on X, and the latter refers to the dissociation energy for the XR → X^+^ + R^−^ reaction. Correlations with *E*_inter_ are poor, however, with correlation coefficients of only 0.44 and 0.56, respectively.

As another interesting quantity, the last column of Table 3 contains the change in the X–Y stretching frequency caused by complexation. This quantity is consistently shifted to the red, consonant with the idea of a weakening X–Y bond. Again there is a clear demarcation between those strongly bonded dyads in the upper regime of the table, with large Δ*ν*, and the smaller red shifts occurring in the lower half of the table.

### Analysis of electron density

Reliable measures of the energy of a particular bond can be derived from AIM analysis of the topology of the electron density. The most useful quantities in this regard all involve the bond critical point, namely its density *ρ*, the Laplacian of the density ∇^2^*ρ*, and the energy density *H*. These three parameters are reported in Table 4 and display many of the same trends as those mentioned above.

**Table 4:** Bond critical point AIM properties of complexes, including electron density (*ρ*), its Laplacian (∇^2^*ρ*), total energy density (*H*), all in au

	*ρ*_1_	*ρ*_2_	*ρ*_1_/*ρ*_2_	∇^2^*ρ*_1_	∇^2^*ρ*_2_	*H*_1_	*H*_2_
Cl_2_	0.1523	0.0459	3.32	−0.0627	0.0993	−0.0796	−0.0039
Br_2_	0.1066	0.0452	2.36	0.0271	0.0734	−0.0403	−0.0046
I_2_	0.0765	0.3014	0.25	0.0488	−0.8436	−0.0224	−0.3881
ClF	0.1462	0.0846	1.73	−0.0370	0.2214	−0.0725	−0.0167
BrF	0.1050	0.0890	1.18	0.0359	0.1953	−0.0392	−0.0245
IF	0.0780	0.0881	0.89	0.0553	0.1957	−0.0239	−0.0283
BrCl	0.1070	0.0537	1.99	0.0276	0.0931	−0.0407	−0.0073
ICl	0.0794	0.0561	1.42	0.0497	0.0774	−0.0246	−0.0109
ClNC	0.1854	0.0181	10.24	−0.1992	0.0606	−0.1217	0.0018
BrNC	0.1179	0.0591	1.99	0.0016	0.1295	−0.0508	−0.0075
INC	0.0830	0.0687	1.21	0.0476	0.1181	−0.0273	−0.0165
ClOH	0.1081	0.0949	1.14	0.0539	0.1750	−0.0374	−0.0218
BrOH	0.0804	0.0953	0.84	0.0704	0.1432	−0.0207	−0.0278
IOH	0.0639	0.0905	0.71	0.0614	0.1322	−0.0149	−0.0309
ClCN	0.0226	0.0950	0.24	0.0517	0.0683	0.0005	−0.0370
BrCN	0.0544	0.1314	0.41	0.0760	−0.0001	−0.0076	−0.0669
ICN	0.0581	0.2292	0.25	0.0613	−0.3508	−0.0119	−0.1854
ClNH_2_	0.0447	0.1339	0.33	0.0802	0.0801	−0.0036	−0.0494
BrNH_2_	0.0494	0.1062	0.47	0.0748	0.0826	−0.0056	−0.0344
INH_2_	0.0469	0.0916	0.51	0.0608	0.0731	−0.0067	−0.0313
ClCF_3_	0.0168	0.1952	0.09	0.0401	−0.2242	0.0010	−0.1269
BrCF_3_	0.0300	0.1381	0.22	0.0550	−0.0465	−0.0010	−0.0678
ICF_3_	0.0444	0.0957	0.46	0.0597	0.0312	−0.0058	−0.0351
ClC_6_F_5_	0.0170	0.1984	0.09	0.0414	−0.2474	0.0010	−0.1379
BrC_6_F_5_	0.0447	0.1262	0.35	0.0711	−0.0160	−0.0043	−0.0591
IC_6_F_5_	0.0525	0.0909	0.58	0.0615	0.0403	−0.0091	−0.0333
ClC_5_H_4_N	0.0081	0.2008	0.04	0.0221	−0.2806	0.0010	−0.1438
BrC_5_H_4_N	0.0162	0.1557	0.10	0.0349	−0.1295	0.0005	−0.0913
IC_5_H_4_N	0.0262	0.1141	0.23	0.0456	−0.0068	−0.0010	−0.0529

The density of the intermolecular X⋯C bond *ρ*_1_ is quite large in the upper portion of Table 4, all larger than 0.06 au, so can be considered largely covalent. This designation is supported by the substantially negative values of *H*_1_. *ρ*_1_ is considerably smaller in the lower segment of the table, although some of these values are in the neighborhood of 0.04 au. In concert with the small negative values of *H*_1_, these bonds would fall into the category of primarily noncovalent but with a significant covalent component. *ρ*_2_ which is related to the internal X–Y bond follows an opposite pattern. Although large enough in the upper part of the table to be deemed covalent, and with a negative H_2_ to support this contention, in most cases, it is the X⋯C bond that is stronger. The *ρ*_1_/*ρ*_2_ ratio is larger than unity in many (but not all) cases, and *H*_1_ is more negative than is *H*_2_. These trends buttress the idea of a high degree of X transfer in these complexes. Again, opposite patterns appear in the lower segment of Table 4: *ρ*_2_ is consistently large, in excess of 0.1 au, and much larger than *ρ*_1_. The *ρ*_1_/*ρ*_2_ ratio is well below unity, highly suggestive of a Y–X⋯C bonding structure containing a covalent Y–X and noncovalent X⋯C halogen bond. *H*_2_ is substantially negative, while *H*_1_ hovers around zero, confirming this sort of arrangement. Note also that once again, the bonding strength parameters for the X⋯C bond diminish in the Cl > Br > I order in the upper portion of the table, while those more weakly held dyads in the lower segment display the opposite sequence.

It might be noted parenthetically that the density of the intermolecular bond critical point follows the normally observed exponential decay with distance. Fig. S1 clearly exhibits this exponential decay, with a correlation coefficient of 0.986. The AIM molecular diagrams and NCI surfaces support the notion of a bond path between the halogen atom and the C of the CLB, as shown for example in Fig. S2, in the exemplary case for ClC_6_F_5_.

The aggregation of CLB with any of the Lewis acids ought to cause a certain amount of charge transfer from the latter to the former. This quantity was assessed as the sum of natural charges on each of the two subunits, and is listed as CT in Table S1. This transfer is quite large in the upper portion of the table, between 0.34 and 0.74 e, in line with the strength of this interaction, and their high degree of halogen transfer. CT diminishes for the more weakly held dyads in the lower portion of the table, more in line with what would be expected for a noncovalent halogen bond. As for the other parameters CT is larger for the lighter X in the upper segment, and for the heavier X in the lower half. CT is fairly closely connected with the total interaction energy. Fig. S3 illustrates an approximately quadratic dependence of *E*_int_ upon CT, with a correlation coefficient of 0.93.

This charge displacement effect weakens with decreasing binding energy (last column of Table S1). The NOCV orbital energies are much larger for strongly bound dyads than for weakly bound ones, with an order of Cl > Br > I in the upper part and the reverse in the lower part. A nearly linear correlation is observed between the NOCV orbital energies and the total interaction energy (Fig. S5, *R*^2^ = 0.97), underscoring the value of NOCV analysis.

As to how the charge redistributes itself upon complexation, several examples are provided in Fig. S4 for dyads of varying binding strength. The patterns are similar, and differ only in magnitude. The primary shifts occur within the Lewis acid, wherein density is shifted away from the electron-donating CLB. Redistributions within the CLB are concentrated around the central C, with decreases on either side of it and an accumulation closer to the nucleus.

**Fig. 5:**
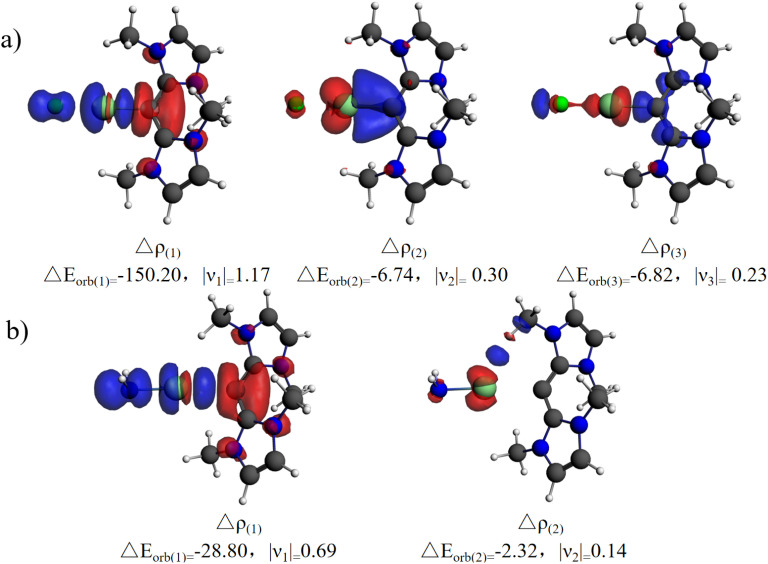
ETS-NOCV deformation density plots for (a) ClF and (b) ClNH_2_ (isosurface value 0.001 au). The major NOCV orbital pairs along with their corresponding orbital interaction energies (Δ*E*_orb(*n*)_, kcal mol^−1^) and eigenvalues(*ν*_n_, au) are shown. The direction of charge flow is indicated as red → blue (red: electron depletion, blue: electron accumulation).

Through ETS-NOCV analysis, one can focus on charge transfer in the context of individual orbitals. Fig. 5 displays the primary NOCV orbitals that mediate this charge transfer for several chlorine-containing Lewis acids. In all systems, the dominant orbital interaction corresponds to electron transfer from the π orbital of the C–C bond in CLB to the σ* antibonding orbital of the Lewis acid 
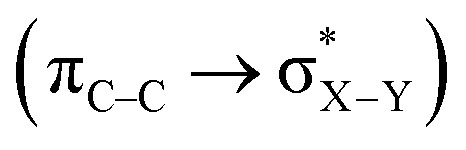
, which is associated with the Δ*ρ*_(1)_ channel in Fig. 5. The deformation density clearly shows electron density displacement from the red regions on the CLB moiety (electron depletion) to the blue regions on the acid (electron accumulation). Upon halogen bond formation, net electron density is transferred from CLB to the halogen acid. The remaining orbital contributions [Δ*ρ*_(*n*)_, *n* ≥ 2] correspond to secondary electron transfer from the halogen acid back to CLB, with their intensities differing significantly between the upper and lower groups of systems.

The ClF system (from the upper group) and the ClNH_2_ system (from the lower group) are taken as examples. In the ClF system, Δ*ρ*_(2)_ and Δ*ρ*_(3)_ correspond to back-donation from the lone pair electrons of the Cl atom to the π* antibonding orbital of the C–C bond in CLB 
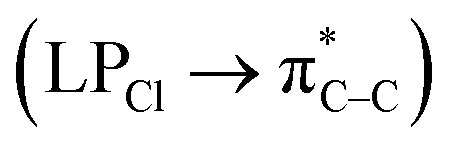
, and from the Cl–F σ bonding orbital to the same π* antibonding orbital of the C–C bond in CLB 
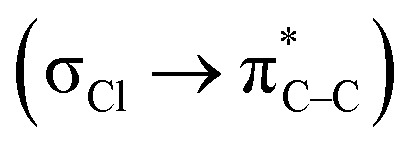
, respectively. Their Δ*E*_orb(*n*)_ are similar, but the electron transfer patterns differ slightly. In Δ*ρ*_(2)_, the carbon atom adjacent to the central carbon exhibits electron density depletion, opposite to the behavior observed in Δ*ρ*_(3)_. Notably, for the ClF system, all three major orbital interactions show signs of participation of the NHC_Me_ ligands of CLB in the back-donation, with the N atoms consistently displaying red (electron depletion) regions. In contrast, the orbital interactions in the ClNH_2_ system are significantly weaker, with only Δ*ρ*_(1)_ showing a dominant charge transfer character. The orbital interaction energy Δ*E*_orb(2)_ for Δ*ρ*_(2)_ is only −2.32 kcal mol^−1^, and accordingly, the isosurface region near the central carbon atom of CLB is hardly observable in Fig. 5, indicating that the secondary charge transfer channel is negligible.

**Fig. 6:**
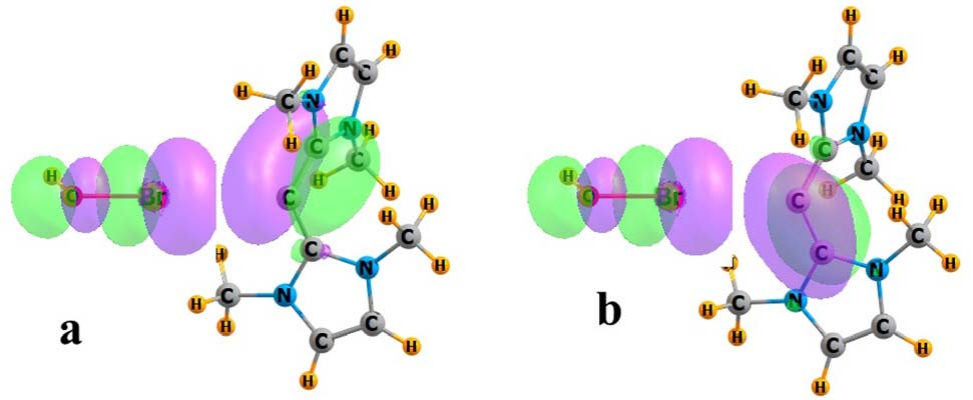
Overlapping (a) and (b) π(CC) → σ*(BrO) NBO orbitals that account for the bulk of interorbital transfer in the complex of CLB with BrOH.

In terms of the NBO analysis of the interorbital charge transfer patterns, the principal localized NBO orbitals involved are portrayed in Fig. 6, taking the complex of CLB with BrOH as an example. σ*(BrO) acts as the primary electron accepting orbital, as would be expected for a halogen bond. The orbitals on CLB that overlap with σ*(BrO) and donate density to it are the two π(CC) orbitals, both involving the central C atom. Within the NBO formalism, the second order perturbation energy *E*_2_ that accompanies each of these two transfers is equal to 29.2 kcal mol^−1^, consistent with the high strength of this XB of 35.5 kcal mol^−1^.

Decomposition of the interaction energy is a commonly applied tool to better understand the nature of the binding. In this study, two methods, namely GKS-EDA and ETS-NOCV, were employed, and both yield consistent trends. The results of a GKS-EDA decomposition are supplied in Table 5 where *E*^ele^ refers to the coulombic interaction between the unperturbed charge distributions of the two subunits, and *E*^pol^ to the stabilizing effect of allowing the two to redistribute the charge. The other attractive term arises from dispersion. The combination of exchange and repulsion leads to the overall repulsive term that prevents the two subunits from coalescing. The systems in the upper half of Table 5 exhibit a high degree of halogen transfer, with large electrostatic and polarization energies (some exceeding 100 or even 500 kcal mol^−1^). Nevertheless, clear trends are apparent: the polarization term often exceeds the electrostatic term, and all components follow the order Cl > Br > I. The systems in the lower half of Table 5, with a lesser degree of halogen transfer, display trends more characteristic of noncovalent halogen bonds: the electrostatic term dominates, accounting for roughly half of the total attraction, with the remainder split approximately evenly between polarization and dispersion. Also characteristic of halogen bonds, all components increase with heavier halogen atoms (Cl < Br < I).

**Table 5:** GKS-EDA energy decomposition of complexes. Electrostatic (*E*^ele^), sum of exchange and repulsion (*E*^ex+rep^), polarization (*E*^pol^), sum of dispersion and electron correlation (*E*^disp^), and total interaction energies (*E*^total^) in kcal mol^−1^ %*E*^ele^, %*E*^pol^, and %*E*^disp^ represent the percentage of each of these three terms relative to their sum

	*E* ^ele^	*E* ^ex+rep^	*E* ^pol^	*E* ^disp^	*E* ^total^	%*E*^ele^	%*E*^pol^	%*E*^disp^
Cl_2_	−147.55	286.73	−212.85	−0.12	−73.79	40.9	59.0	0.0
Br_2_	−120.57	208.96	−137.43	−8.20	−57.25	45.3	51.6	3.1
I_2_	−82.52	147.12	−97.10	−11.41	−43.90	43.2	50.8	6.0
ClF	−138.23	258.29	−185.87	−7.74	−73.55	41.7	56.0	2.3
BrF	−117.80	191.72	−123.60	−10.74	−60.42	46.7	49.0	4.3
IF	−85.39	138.40	−93.48	−9.92	−50.40	45.2	49.5	5.3
BrCl	−121.00	206.09	−136.28	−8.21	−59.39	45.6	51.3	3.1
ICl	−86.90	147.75	−101.39	−9.55	−50.09	43.9	51.3	4.8
ClNC	−276.33	518.10	−534.62	−32.37	−325.22	32.8	63.4	3.8
BrNC	−135.94	228.62	−180.57	19.30	−68.58	45.7	60.8	−6.5
INC	−92.27	153.24	−122.04	6.75	−54.32	44.5	58.8	−3.3
ClOH	−96.78	185.92	−106.25	−23.14	−40.25	42.8	47.0	10.2
BrOH	−85.04	143.72	−74.02	−20.14	−35.48	47.5	41.3	11.2
IOH	−66.95	112.76	−63.44	−15.26	−32.89	46.0	43.6	10.5
ClCN	−16.64	24.00	−6.35	−7.58	−6.57	54.4	20.8	24.8
BrCN	−54.51	86.06	−39.99	−13.81	−22.25	50.3	36.9	12.8
ICN	−60.99	98.71	−59.53	−9.24	−31.04	47.0	45.9	7.1
ClNH_2_	−33.70	66.23	−18.00	−23.92	−9.38	44.6	23.8	31.6
BrNH_2_	−47.47	83.77	−28.54	−22.84	−15.08	48.0	28.9	23.1
INH_2_	−46.69	82.55	−35.77	−18.51	−18.41	46.2	35.4	18.3
ClCF_3_	−10.11	16.37	−3.89	−5.19	−2.82	52.7	20.3	27.1
BrCF_3_	−24.85	40.54	−14.08	−10.07	−8.46	50.7	28.7	20.6
ICF_3_	−43.14	72.68	−37.76	−10.25	−18.47	47.3	41.4	11.3
ClC_6_F_5_	−10.19	17.04	−2.00	−7.56	−2.71	51.6	10.1	38.3
BrC_6_F_5_	−41.55	69.91	−24.26	−17.79	−13.69	49.7	29.0	21.3
IC_6_F_5_	−52.75	90.76	−46.37	−14.59	−22.95	46.4	40.8	12.8
ClC_5_H_4_N	−3.56	6.23	−0.37	−2.46	−0.17	55.7	5.8	38.5
BrC_5_H_4_N	−10.68	17.96	−3.01	−6.73	−2.45	52.3	14.7	33.0
IC_5_H_4_N	−21.91	37.70	−13.22	−10.33	−7.76	48.2	29.1	22.7

The ETS-NOCV results (Table S2) further support the above conclusions. In the upper half systems, electrostatics and orbital interactions jointly dominate; in the lower half systems, electrostatics plays a more prominent role. The overall trends are largely consistent with those obtained from GKS-EDA.

## Discussion

Carbones are a class of extremely strong Lewis bases. Tonner *et al.*^66^ demonstrated that they possess not only a very high first proton affinity but also a remarkably high second proton affinity. In contrast, while carbenes also have a high first proton affinity, their second proton affinity is much lower than that of carbones. While two successive protons can each attach to a separate lone pair of the carbone C, carbenes possess only a single pair. The second proton leads to a drastic change in bonding toward a CH_2_^2+^ cation with the remainder of the molecule acting as electron donor. The carbone center of the CLB molecule contains a strongly negative region of its electrostatic potential directed along its C–C–C bisector, and functions as a powerful Lewis base, attracting the positive σ-hole of any number of halogen-containing molecules as Lewis acids. These interactions exhibit some unique properties. In the first place, there is a very wide spectrum of interaction strength, from as little as less than 1 kcal mol^−1^, all the way up to nearly 80 kcal mol^−1^. The bridging halogen atom can transfer across to the CLB to varying degrees, and in some cases this transfer is nearly complete. There is a tight correlation noted between the degree of halogen transfer and the strength of the binding. Grabowski^67^ found in his study of tetrel bonding systems that the formation of a σ-hole bond is essentially a prelude to the subsequent S_N_2 reaction, inducing the same electron redistribution as that in a covalent bond-forming reaction. This further confirms that, under sufficiently strong halogen bonding, the system may undergo bond breaking and formation, manifesting as halogen atom transfer.

The dyads separate themselves into two broad categories. The more weakly bound, with binding energies less than 20 kcal mol^−1^, can be classified as traditional halogen bonds. The binding rises steeply along with the size of the X atom, and the depth of its σ-hole. There is a red shift of the internal X–Y stretching frequency of as much as 300 cm^−1^, as this bond weakens. The second category of interactions is considerably stronger and is accompanied by a substantial amount of halogen transfer, along with the accompanying formation of a covalent C–X bond. The binding energies vary between 20 and 45 kcal mol; interaction energies approach and even exceed 80 kcal mol^−1^. The central X atom undergoes a very substantial displacement toward the CLB, stretching the X–Y bond by as much as 0.5 Å, and in some cases can be considered fully transferred. Opposite to the pattern in noncovalent XBs, the bond strengths in these complexes diminish as the X atom grows larger, even though its σ-hole becomes more positive.

Although there is an undeniable amount of halogen transfer in the first category of more weakly held complexes, this displacement is only moderate, with stretches of the X–Y bond less than 0.2 Å. The transfer parameter *t* remains less than 0.6 where a value of 1 defines half transfer. The second and more strongly bound grouping of dyads has considerably larger transfer parameters, some as large as 4 or 5, essentially transforming into CLBX^+^···Y^−^ ion pairs. Another distinguishing feature between the two sorts of dyad is the composition of their interaction energy. The more weakly bonded complexes, with only moderate amounts of halogen transfer, are held together largely by coulombic forces, typical of halogen bonds. This composition shifts for the more tightly held structures where polarization becomes more important, in some cases exceeding electrostatics. These stronger complexes also contain a lesser percentage contribution from dispersion.

It is worthwhile to elucidate the reasons that a given Lewis acid falls into one category or the other. As a broad observation, the acids that are more prone to dissociate are the dihalogens, as well as XNC and XOH. These substituents are generally electron-withdrawing. The dissociation energies of this group of acids, *i.e.* resistance to loss of halogen, tend to be less than 300 kcal mol^−1^, although there are exceptions. The second category, more resistant to loss of a halogen, tends to have dissociation energies above this threshold, but again with some exceptions, so this is not a hard and fast rule. The substituents in this category are generally less electron-withdrawing, and in some cases electron-donating. As a second issue, inspection of the upper section of Table 2 reveals that the degree of halogen transfer is largest for Cl and then diminishes for Br, and further for I. However, this pattern reverses for the lower section where the transfer parameter is smaller, but rises a bit for larger X atoms. This latter pattern is consistent with the idea that smaller dissociation energies might work to facilitate a certain amount of transfer to the base. Indeed, this pattern noted for those in the second section, with the lesser degree of halogen transfer, is consistent with standard expectations for halogen bonding, *viz.* stronger for heavier X (see Table 3).

The carbone under consideration here serves as a particularly effective electron donor. In order to place this potency into some perspective, it can be compared with a number of other Lewis bases. The *V*_s,min_ at the donor atoms (C/N/O) of several such bases (NH_3_, NMe_3_, C_2_H_5_O^−^, CMe_2_, and CMe_3_^−^) are listed in the first row of Table 6 where they can be compared with the −36.4 kcal mol^−1^ of CLB. The neutral N bases NH_3_ and NMe_3_ have minima of roughly this same value, which is slightly surpassed by that of the carbene CMe_2_ which contains a single C lone pair. This quantity is of course far more negative for the two anions C_2_H_5_O^−^ and CMe_3_^−^. The succeeding rows of Table 6 compare the interaction energies of each of these bases with a series of Br-containing Lewis acids. These data further amplify the power of the CLB carbone as a Lewis base. Its interaction energy with each of these acids is considerably larger than most of the other bases. They easily surpass the two N bases, and the carbene, despite comparable *V*_s,min_. Indeed, the neutral CLB carbone leads to a stronger bond than even the ethoxide anion in several cases, with a *V*_s,min_ only 23% that of the anion. It is only the CMe_3_^−^ anion that provides a consistently stronger interaction. These comparisons illustrate that the strong electron-donating capacity of CLB stems not from electrostatics but from the unique σ/π dual lone-pair structure of its carbone center. CLB combines near-anionic basicity with the sensitivity of a neutral base toward different Lewis acids. Even without the amplification effect of a full negative charge, this neutral molecule serves as a remarkably powerful electron donor.

**Table 6:** Minimum of the MEP (*V*_s,min_, kcal mol^−1^) on the 0.001 au isodensity surface near the C/N/O atom of the Lewis base, and interaction energy of various Lewis bases with Br-containing Lewis acids; all quantities in kcal mol^−1^

	CLB	NH_3_	NMe_3_	C_2_H_5_O^−^	CMe_2_	CMe_3_^−^
*V*_s,min_	−36.4	−38.7	−32.4	−158.5	−42.0	−139.5
Br_2_	−57.24	−12.23	−10.99	−36.21	−21.85	−66.86
BrF	−60.42	−20.45	−16.26	−36.38	−28.60	−63.38
BrNC	−68.58	−13.26	−16.12	−47.23	−26.48	−76.94
BrOH	−35.48	−10.16	−9.25	−25.51	−20.87	−46.98
BrCN	−22.25	−6.19	−7.61	−36.98	−13.55	−53.53
BrNH_2_	−15.07	−3.87	−1.18	−15.10	−7.34	−28.77
BrCF_3_	−8.46	−3.44	0.15	−20.38	−3.17	−28.19
BrC_6_F_5_	−13.68	−3.42	−1.17	−26.75	−0.83	−41.16
BrC_5_H_4_N	−2.45	−1.58	2.65	−19.48	−6.08	−26.07

As for the underlying nature of the bonding in these complexes, a prime feature is a large amount of charge transfer. Even in those systems where the halogen transfer is minimal, the amount of charge shifted from the CLB to the Lewis acid reaches up toward 0.3–0.5 e. This transfer is dominated by the overlap between the C–C π-bonding orbitals of the CLB and the σ*(XR) antibonding orbital of the acid. The former orbitals are characteristic of the bent C=C=C electronic structure associated with the bonding scheme connected with the carbone base. An earlier study by Tonner and Frenking^31^ of carbone compounds found a good deal of electron donation of electron density from ligand L to the central carbon. This process results in a net increase in the central carbon's electron density and results in an overall exceptionally high basicity and coordination capability. For carbone compounds such as CLB, the strength of halogen-bond interactions likely depends on whether a similar electron-enhancement mechanism can be effectively triggered, rather than being determined solely by initial electrostatic attraction.

There is some precedent in the literature for the idea of halogen transfer across a halogen bond, although the bulk of this work might be better characterized as partial transfer at best, in some cases simply stretches of the X–Y bond. A number of early calculations^68–72^ focused primarily on Cl, that could transfer across to a cyano group for example. Other work noted the partial transfer of Br to a heterocyclic carbene or between P and F centres.^73^ The I atom was also shown to be amenable to transfer between two N atoms.^74–77^ Another study^78^ presented the idea that such a transfer might be promoted by the cooperativity associated with a third entity. In the case of an ionic system, *e.g.* (AX⋯B)^+^, transfer is typically facilitated since this process will simply convert one ion–neutral pair to another, rather than the creation of a high-energy ion pair from a neutral pair in the case of a neutral system.^79–96^ Previous calculations by this group^97^ have elucidated the governing principles of halogen transfer in the context of a cationic system where the X^+^ is shifting between a pair of neutral molecules, and found strong similarities with proton transfer. These symmetric transfer potentials are of single-well character when the halogen bond is short, but evolve to double wells for longer intermolecular separation. The phenomenon of halogen transfer is also consistent with our own very recent research^98^ involving Be(CO)_3_, where complexes with XF and XCl exhibit strong interactions exceeding 40 kcal mol^−1^ and significant X-atom displacement.

The results presented here underscore the idea that halogen-bond strength is not solely determined by the static σ-hole potential. As another example, Wang *et al.*^99^ observed a so-called “blue-shifting” XB where, counterintuitively, the R–X bond shortened upon XB formation – a sign that factors beyond a simple σ-hole depth were influencing the interaction. However, no such blue-shifting XB is observed in the CLB halogen-bonded complexes examined here. Likewise, Zheng *et al.*^100^ demonstrated a pronounced cooperative effect in a multi-component system: the formation of an F–Ag coordination bond strengthened a neighboring X⋯Br XB *via* mutual polarization, illustrating how an external perturbation can enhance a XB beyond static expectations. These examples and the present data emphasize that an XB can range from largely electrostatic, *i.e.* adherence-to-σ-hole principles, to highly polarized, partially covalent engagements, depending on the ability of the donor to undergo electronic reorganization and release of its halogen atom.

## Conclusions

The carbone center offers an especially strong site for interaction with a Lewis acid, much more so than would be predicted by its electrostatic potential, which has a minimum of magnitude slightly smaller than that of NH_3_, but bonds much more strongly. The attraction of this carbone center for the halogen atom of the Lewis acid is powerful enough as to induce a high degree of halogen transfer in many of these dyads. The interaction energies rise up to more than 70 kcal mol^−1^ in several cases. Even after this quantity is reduced by consideration of monomer deformations within the complex, the binding energies are still quite large, many higher than 40 kcal mol^−1^. The complexes span a spectrum not only of energies, but also of the degree of halogen transfer, which is magnified for the more strongly bound dyads. There appears to be a dividing line of sorts: the more weakly bound complexes manifest most of the characteristics of a halogen bond, including a large proportion of electrostatic interaction, while there is a strong element of covalency for those that are more tightly bound. This strong interaction, which transcends the limits of electrostatics, highlights the great potential of the carbone center as an electron donor for constructing functional supramolecular systems, a concept that warrants further validation across a broader range of acceptor systems.

## Author contributions

S. Li: methodology, investigation, data curation, preparation of figures in the appendix, writing – original draft, and manuscript formatting. H. Zhou: writing – review and editing. Q. Li: conceptualization, methodology, writing – review and editing, supervision, funding acquisition, writing – review and editing, and resources. S. Scheiner: writing – review and editing, preparation, data verification and validation.

## Conflicts of interest

The authors declare no conflict of interest.

## Supplementary Material

SC-017-D6SC02210C-s001

## Data Availability

Supplementary information (SI): the C⋯X electron density *vs.* bond length correlation, AIM and NCI plots for the ClC_6_F_5_ system, charge transfer *vs.* interaction energy correlation, electron density difference maps, NOCV orbital energy *vs.* interaction energy correlation (Fig. S1–S5), charge transfer, the major NOCV orbital energies, and ETS-NOCV interaction energy components (Table S1–S2), along with the Cartesian coordinates of all monomers and complexes. See DOI: https://doi.org/10.1039/d6sc02210c.
