# Supplementary material for: Strong interactions between carbones and halogen atomic centers
Source: Chem Sci. 2026 Jun 19;17(31):14950–62. doi: 10.1039/d6sc02210c (PMC13289791; doi:10.1039/d6sc02210c)
Supplement: Supplementary file 1 [file SC-017-D6SC02210C-s001.pdf]

Supplementary Information (SI) for Chemical Science.
This journal is © The Royal Society of Chemistry 2026

Supporting Information

Strong Interactions between Carbones and Halogen Atomic Centers

Shunhua Li,[a] Hangyu Zhou,[a] Qingzhong Li,[a,*] Steve Scheiner*,[b]

[a] The Laboratory of Theoretical and Computational Chemistry, School of Chemistry and Chemical Engineering, Yantai University, Yantai 264005, P. R. China.
[b] Department of Chemistry and Biochemistry, Utah State University, Logan, UT 84322-0300, USA

Corresponding authors: Qingzhong Li and Steve Scheiner
E-mails: lqz@ytu.edu.cn; steve.scheiner@usu.edu

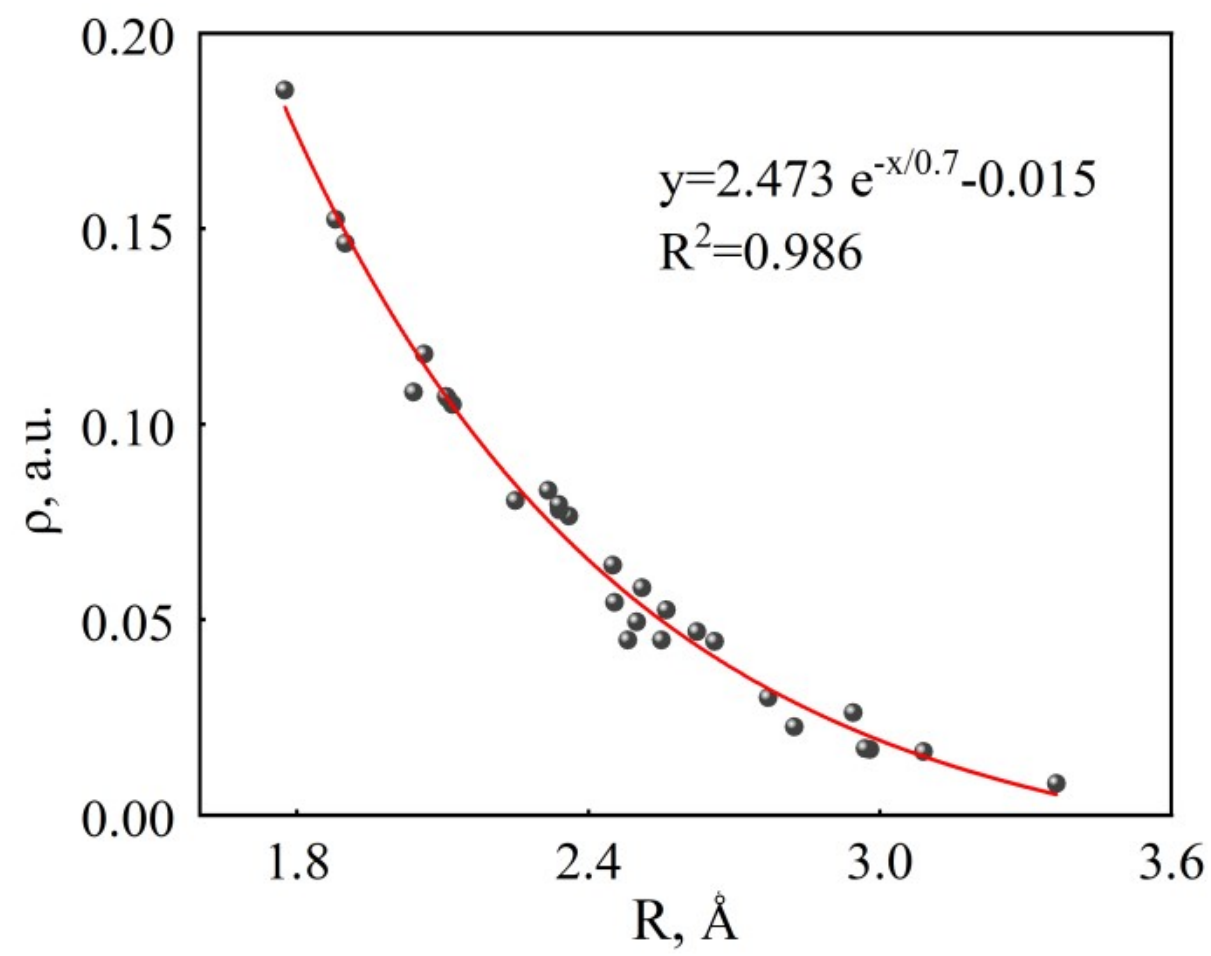

Fig. S1. Relationship between the C···X distance (R) and the electron density (ρ) at the C···X bond critical point.

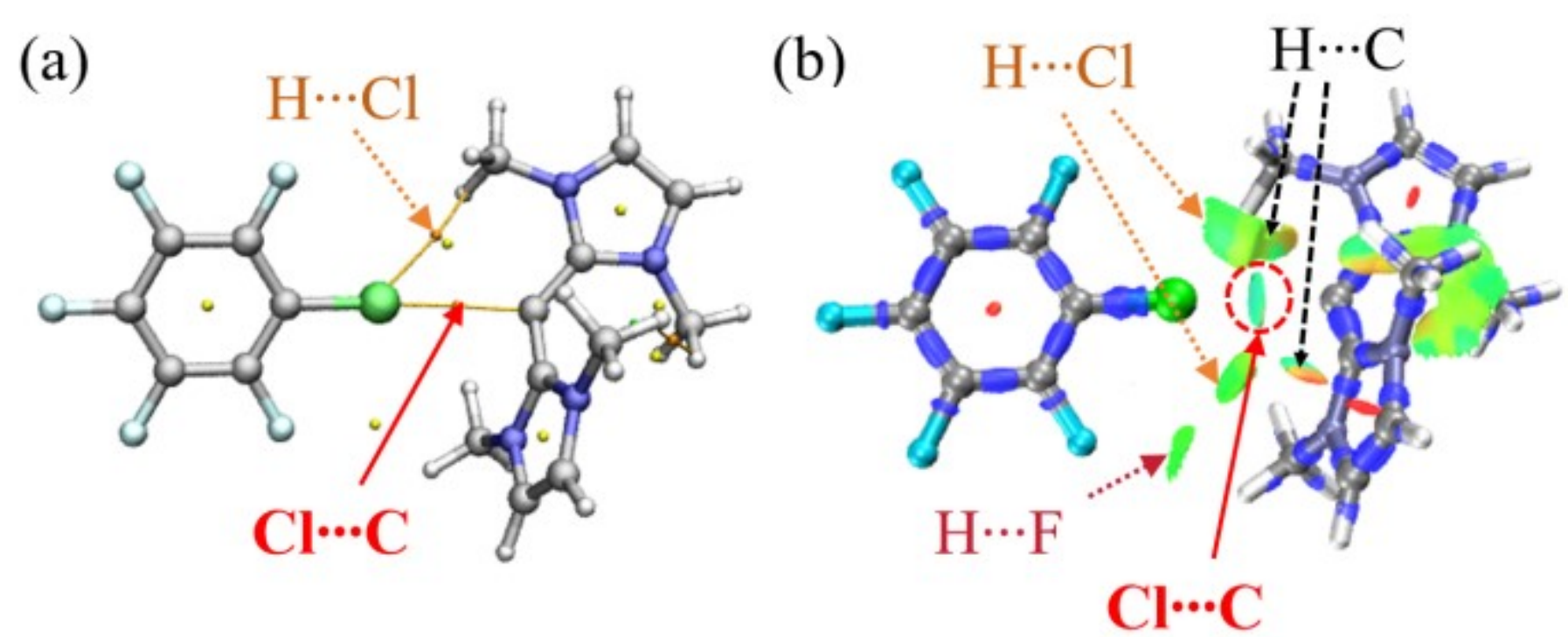

Fig. S2. (a) AIM plot and (b) NCI plot for the CLB···ClC_6F_5 complex. In the NCI plot, blue, green, and red regions correspond to strong attractive, weak attractive, and strong repulsive interactions, respectively.

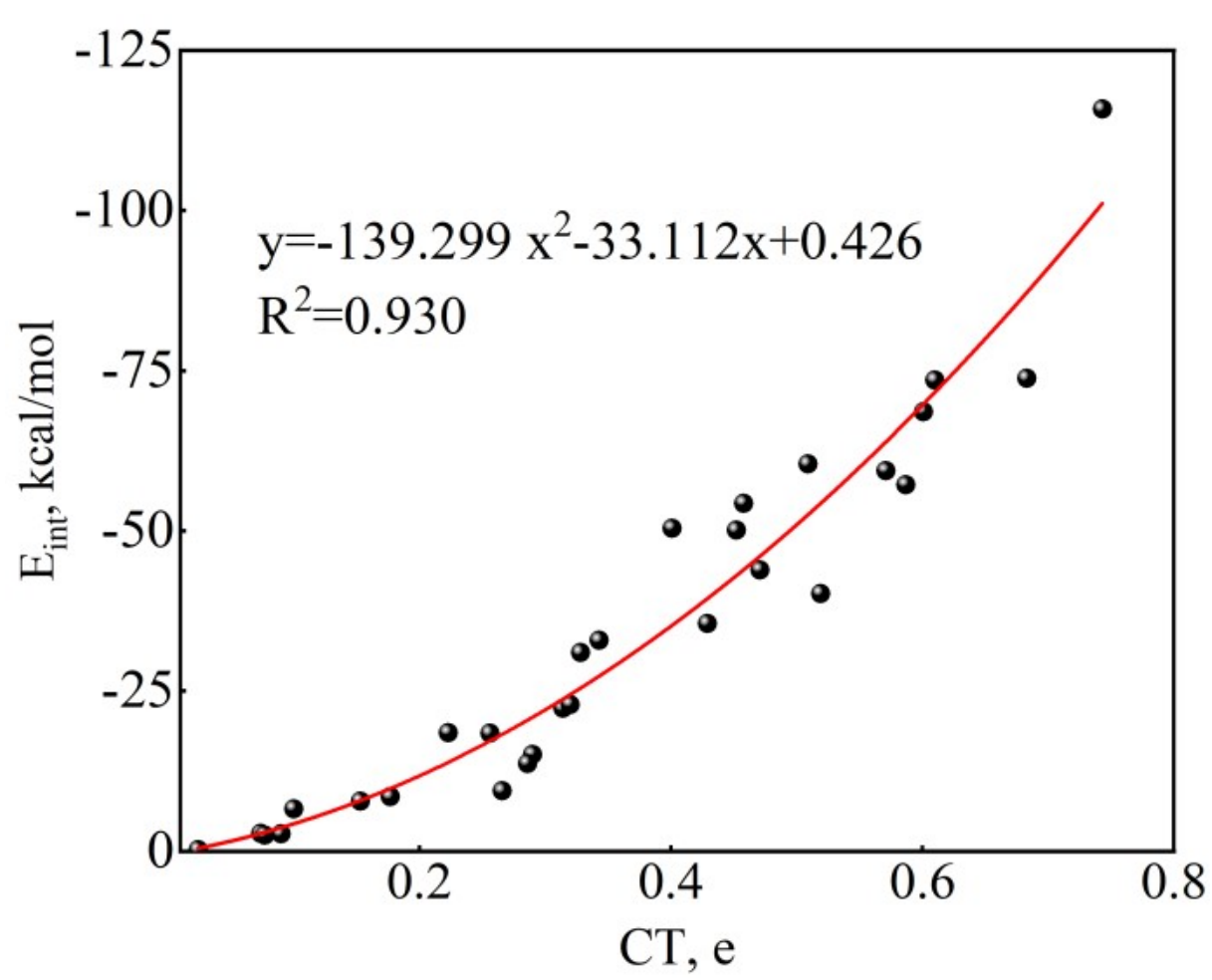

Fig. S3. Correlation between charge transfer (CT) and interaction energies (E_{int}).

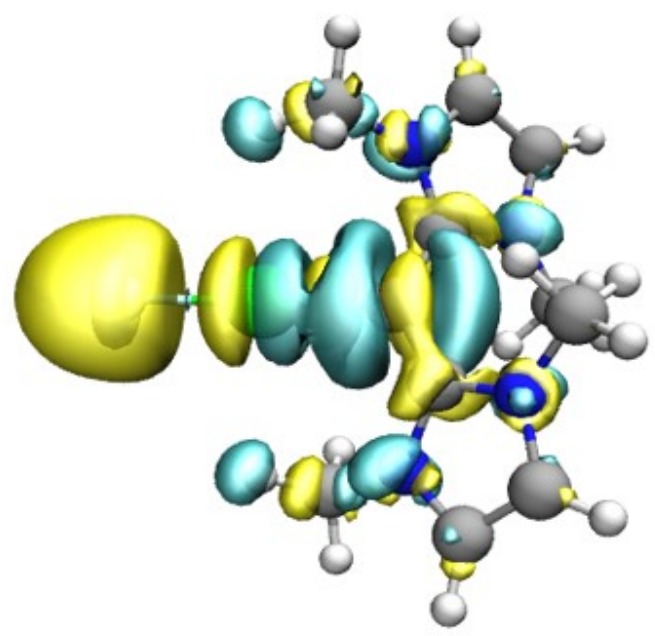

$C(NHC_{Me})_2$-ClF

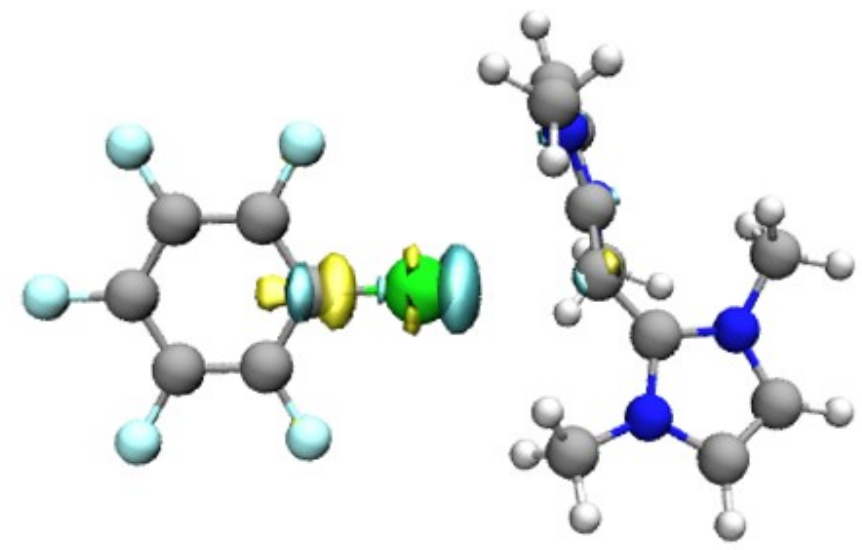

$C(NHC_{Me})_2$-C_6F_5Cl

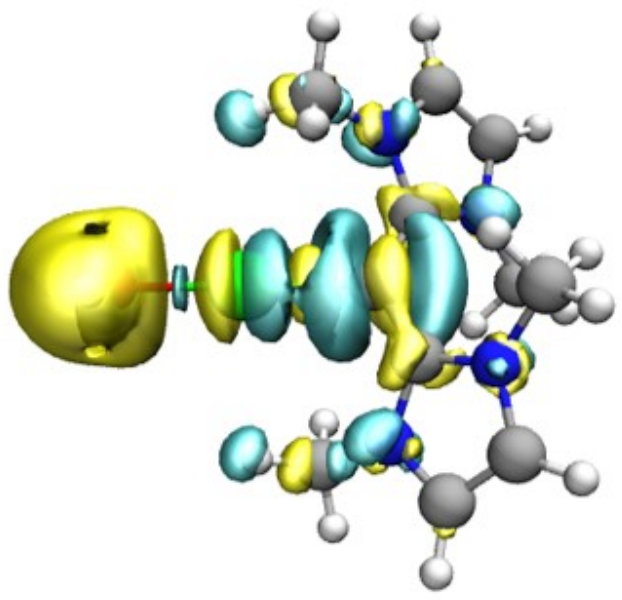

$C(NHC_{Me})_2$-HOCl

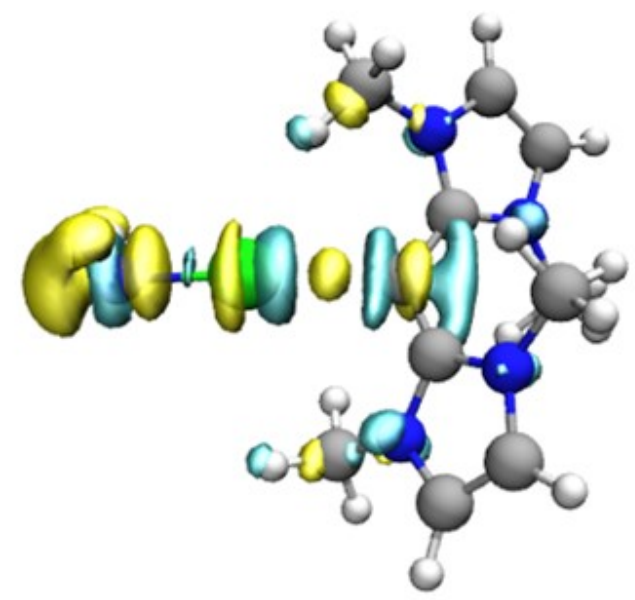

$C(NHC_{Me})_2$-NH_2Cl

Fig. S4. Electron density changes upon complex formation. The isosurface level is set at ±0.002 a.u. Yellow regions (+0.002 a.u.) indicate an increase in electron density, whereas blue regions (−0.002 a.u.) indicate a decrease in electron density.

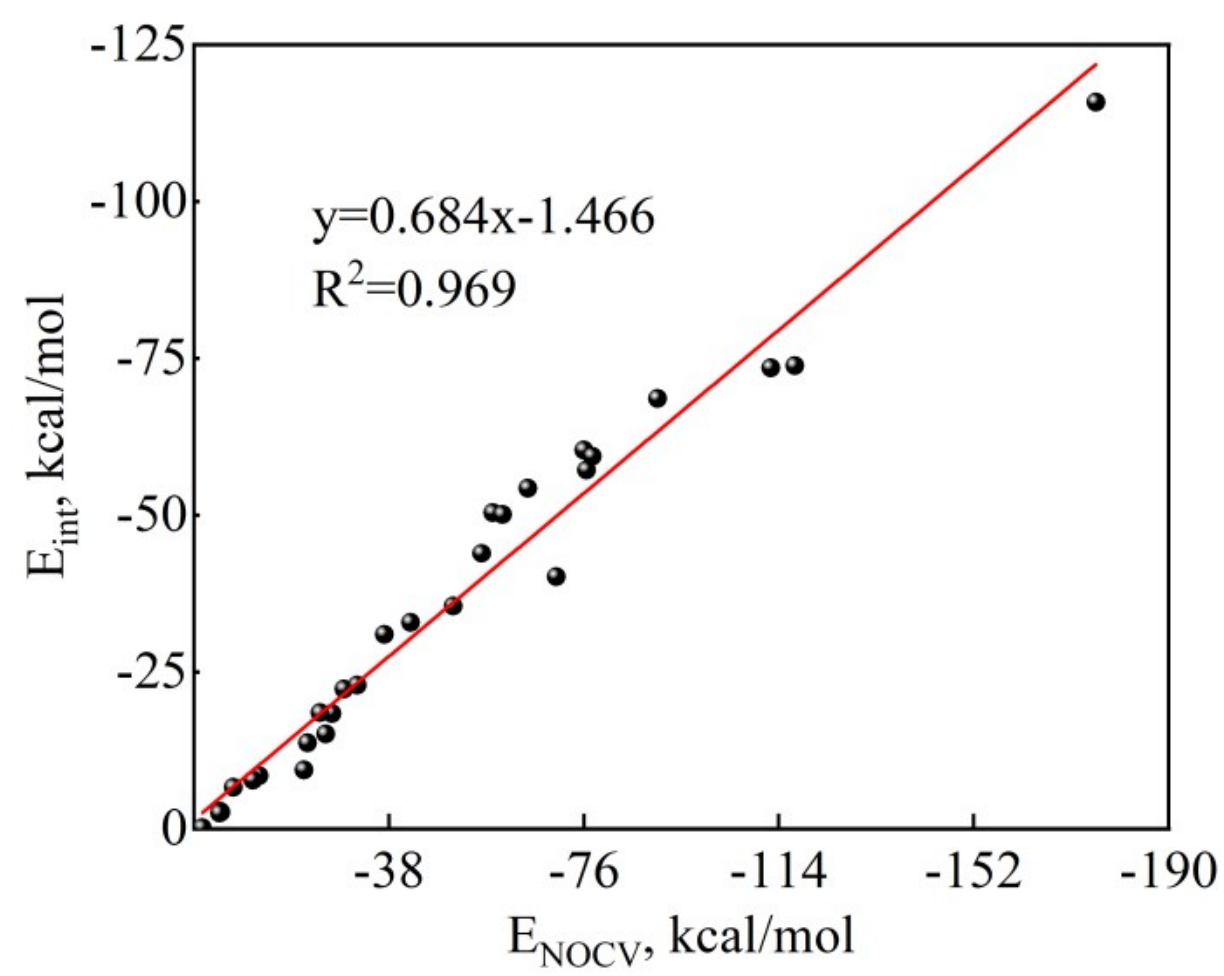

Fig. S5. Correlation between NOCV orbital energies (E_{NOCV}) and interaction energies (E_{int}).

Table S1 Charge transfer from CLB to XY (CT, e) and the major NOCV orbital energies (E_{nocv}, kcal/mol) from π_{C-C} into σ^*_{X-Y}.

	CT	E_{nocv}		CT	E_{nocv}
Cl_2	0.683	-117.08	ClCN	0.100	-7.69
Br_2	0.587	-76.55	BrCN	0.314	-29.25
I_2	0.471	-56.07	ICN	0.328	-37.09
ClF	0.610	-112.44	$ClNH_2$	0.266	-21.49
BrF	0.509	-76.02	$BrNH_2$	0.290	-25.69
IF	0.401	-58.26	INH_2	0.256	-26.94
BrCl	0.571	-77.60	$ClCF_3$	0.074	-5.11
ICl	0.452	-60.13	$BrCF_3$	0.177	-12.71
ClNC	0.743	-175.82	ICF_3	0.223	-24.57
BrNC	0.601	-90.33	ClC_6F_5	0.090	-5.30
INC	0.458	-65.11	BrC_6F_5	0.286	-22.13
ClOH	0.519	-70.61	IC_6F_5	0.320	-31.83
BrOH	0.429	-50.50	ClC_5H_4N	0.024	-1.63
IOH	0.343	-42.21	BrC_5H_4N	0.077	-4.96
			IC_5H_4N	0.153	-11.50

Table S2 ETS-NOCV interaction energy components, all in kcal/mol.

	$\triangle E_{Pauli}$	$\triangle E_{elstat}$	$\triangle E_{disp}$	$\triangle E_{orb}$	$\triangle E_{int}$
Cl_2	264.00	-150.88	-4.98	-187.00	-78.86
Br_2	185.77	-120.47	-6.30	-124.31	-65.30
I_2	143.38	-100.43	-7.59	-86.87	-51.52
ClF	238.36	-140.27	-4.73	-172.42	-79.05
BrF	171.32	-117.39	-5.98	-116.99	-69.04
IF	138.10	-104.23	-7.24	-84.91	-58.29
BrCl	183.53	-120.91	-6.14	-123.93	-67.45
ICl	145.72	-106.03	-7.36	-90.16	-57.85
ClNC	828.33	-294.15	-6.75	-786.17	-258.73
BrNC	207.62	-136.46	-6.08	-140.64	-75.55
INC	154.17	-113.10	-7.34	-95.14	-61.41
ClOH	167.23	-97.83	-4.88	-110.70	-46.17
BrOH	125.20	-84.28	-6.02	-78.94	-44.04
IOH	109.14	-80.33	-7.19	-62.53	-40.91
ClCN	38.36	-27.20	-4.42	-19.47	-12.72
BrCN	73.61	-53.50	-5.87	-44.50	-30.26
ICN	95.13	-72.10	-7.20	-54.50	-38.67
$ClNH_2$	56.65	-33.28	-4.20	-33.37	-14.20
$BrNH_2$	70.41	-46.38	-5.33	-40.93	-22.23
INH_2	76.86	-54.55	-6.44	-41.28	-25.41
$ClCF_3$	14.28	-10.21	-3.83	-7.14	-6.90
$BrCF_3$	33.92	-24.57	-5.41	-19.27	-15.32
ICF_3	68.13	-50.30	-7.05	-37.16	-26.38
ClC_6F_5	14.85	-10.09	-4.46	-7.74	-7.44
BrC_6F_5	58.70	-40.69	-6.68	-33.61	-22.27
IC_6F_5	85.63	-62.22	-7.79	-46.92	-31.30
ClC_5H_4N	5.74	-3.39	-3.17	-2.52	-3.35
BrC_5H_4N	14.85	-10.35	-4.92	-7.85	-8.26
IC_5H_4N	33.94	-24.68	-6.54	-17.25	-14.54

Coordinates of Monomers and Dyads

Monomers

Cl_2

Cl	0.00000000	0.00000000	1.00828300
Cl	0.00000000	0.00000000	-1.00828300

Br_2

Br	0.00000000	0.00000000	1.15897700
Br	0.00000000	0.00000000	-1.15897700

I_2

I	0.00000000	0.00000000	1.34645100
I	0.00000000	0.00000000	-1.34645100

ClF

F	0.00000000	0.00000000	-1.08256000
Cl	0.00000000	0.00000000	0.57312000

BrF

F	0.00000000	0.00000000	-1.42363200
Br	0.00000000	0.00000000	0.36607700

IF

F	0.00000000	0.00000000	-1.65784300
I	0.00000000	0.00000000	0.28152100

BrCl

Br	0.00000000	0.00000000	0.70886600
Cl	0.00000000	0.00000000	-1.45942900

ICl

Cl	0.00000000	0.00000000	-1.77746600
I	0.00000000	0.00000000	0.57013000

ClNC

N	0.00000000	0.00000000	-0.68772900
C	0.00000000	0.00000000	-1.87624800
Cl	0.00000000	0.00000000	0.94538800

BrNC

N	0.00000000	0.00000000	-1.16301700
C	0.00000000	0.00000000	-2.35094400
Br	0.00000000	0.00000000	0.63562200

INC

N	0.00000000	0.00000000	-1.48855600
C	0.00000000	0.00000000	-2.67610600
I	0.00000000	0.00000000	0.49955700

HOCl

O	0.03683500	1.11458600	0.00000000
H	-0.92087600	1.32005600	0.00000000
Cl	0.03683500	-0.60216100	0.00000000

HOBr

O	0.02173500	1.47316300	0.00000000
H	-0.93461000	1.68061800	0.00000000
Br	0.02173500	-0.38474100	0.00000000

HOI

O	0.01531300	1.71843900	0.00000000
H	-0.93406700	1.95215500	0.00000000
I	0.01531300	-0.29622000	0.00000000

ClCN

C	0.00000000	0.65333200	0.00000000
N	-0.00321800	1.82134400	0.00000000
Cl	0.00132500	-0.98055300	0.00000000

BrCN

C	-0.00055900	1.13886200	0.00000000
N	0.00047900	2.30694800	0.00000000
Br	0.00000000	-0.65662300	0.00000000

ICN

C	-0.00139700	1.48060600	0.00000000
N	0.00119700	2.64918700	0.00000000
I	0.00000000	-0.51750800	0.00000000

NH_2Cl

N	-0.04461000	1.14180600	0.00000000
H	0.53531500	1.37537400	0.81699900
H	0.53531500	1.37537400	-0.81699900
Cl	-0.04461000	-0.63196400	0.00000000

NH_2Br

N	-0.02678600	1.52429400	0.00000000
H	0.56250500	1.74345700	0.81474200
H	0.56250500	1.74345700	-0.81474200
Br	-0.02678600	-0.40448500	0.00000000

NH_2I

N	-0.01893600	1.79165800	0.00000000
H	0.56808000	2.02032700	0.81375900
H	0.56808000	2.02032700	-0.81375900
I	-0.01893600	-0.31287300	0.00000000

$ClCF_3$

C	-0.35450500	0.00009800	0.00000000
F	-0.81838700	-0.62970800	1.09093500
F	-0.81838700	-0.63021500	-1.09064300
F	-0.81868800	1.25970100	-0.00029300
Cl	1.42507000	0.00008200	0.00000000

$BrCF_3$

C	-0.82488200	0.00000000	0.00009200
F	-1.28647600	1.09270700	0.62692800
F	-1.28647600	-1.08946400	0.63254400
F	-1.28611200	-0.00324300	-1.25971200
Br	1.13373900	0.00000000	0.00004600

ICF_3

C	-1.19722400	-0.00004800	0.00000100
F	-1.66372300	0.62916200	-1.09183600
F	-1.66371800	0.63110700	1.09071400
F	-1.66377500	-1.26021600	0.00112300
I	0.98310000	-0.00000300	0.00000000

C_6F_5Cl

C	1.68788900	0.00000900	-0.00001600
C	0.98794300	-1.21027700	-0.00003000
C	-0.40973400	-1.20651100	0.00000100
C	-1.12291800	0.00001500	-0.00002000
C	-0.40973700	1.20649600	-0.00003800
C	0.98797600	1.21027100	-0.00000400
F	1.65960100	-2.37136600	0.00002300
F	3.02775500	-0.00004300	0.00000600
F	1.65956500	2.37139600	0.00001200
F	-1.06011100	2.37818800	0.00001600
F	-1.06016500	-2.37816500	0.00000000
Cl	-2.84519500	-0.00000700	0.00000800

C_6F_5Br

C	2.17727000	0.00000600	-0.00000500
C	1.47754600	-1.21034300	-0.00001100
C	0.07962900	-1.20572700	0.00000000

C	-0.63150500	0.00002200	-0.00000700
C	0.07963400	1.20571400	-0.00001500
C	1.47759500	1.21033300	-0.00000100
F	2.14982400	-2.37125400	0.00000500
F	3.51699000	-0.00005800	0.00000400
F	2.14980200	2.37127800	0.00000400
F	-0.56606400	2.38068200	0.00000500
F	-0.56614100	-2.38064800	0.00000200
Br	-2.51773500	-0.00000100	0.00000100

C_6F_5I

C	2.59669600	0.00000500	-0.00000600
C	1.89693000	-1.21030100	-0.00001600
C	0.49900100	-1.20326600	0.00000100
C	-0.21460900	0.00002200	-0.00000900
C	0.49901000	1.20325600	-0.00002000
C	1.89697800	1.21029300	0.00000000
F	2.57020200	-2.37110800	0.00000700
F	3.93633100	-0.00005700	0.00000500
F	2.57018600	2.37113000	0.00000600
F	-0.14187400	2.38294900	0.00000600
F	-0.14194700	-2.38291700	0.00000400
I	-2.30528500	0.00000000	0.00000100

C_5H_4NCl

C	1.59299600	-1.14289900	0.00002500
C	0.19610800	-1.20569200	-0.00003900
C	-0.50566500	0.00018600	-0.00014900
C	0.19628900	1.20585300	-0.00004300
C	1.59321900	1.14270500	0.00003000
N	2.29760400	-0.00013000	0.00002000
H	2.17555700	-2.06950300	0.00008400
H	-0.32568000	-2.16271300	0.00000200
H	-0.32511700	2.16308700	-0.00003900
H	2.17591500	2.06923200	0.00012600
Cl	-2.24832900	-0.00000600	0.00004400

C_5H_4NBr

C	-2.20103900	-1.14254200	-0.00000900
C	-0.80350700	-1.20595700	0.00002200
C	-0.10342700	0.00015900	0.00007700
C	-0.80365500	1.20606700	0.00003500
C	-2.20123200	1.14238700	-0.00001400
N	-2.90542100	-0.00010100	-0.00003000
H	-2.78269200	-2.06971000	-0.00000100
H	-0.28579800	-2.16506000	-0.00001200

H	-0.28621800	2.16534600	0.00000100
H	-2.78297400	2.06950300	-0.00004400
Br	1.80436500	-0.00000100	-0.00001100

C_5H_4NI

C	-2.66511500	-1.14205900	-0.00002500
C	-1.26671500	-1.20508300	0.00004000
C	-0.56297500	0.00016400	0.00012100
C	-1.26691300	1.20518300	0.00004100
C	-2.66535100	1.14190500	-0.00001900
N	-3.36946000	-0.00011800	-0.00001800
H	-3.24627600	-2.06988300	-0.00009500
H	-0.75563000	-2.16777800	-0.00004000
H	-0.75604900	2.16802100	-0.00005100
H	-3.24662800	2.06965900	-0.00010700
I	1.55006100	0.00000300	-0.00001000

$C(NHC_{Me})_2$

C	0.00000800	-0.79373600	-0.00006300
C	3.09217900	0.97713200	0.69153300
C	3.53175400	-0.06707100	-0.05856500
H	3.64585500	1.77533000	1.17289200
H	4.53778200	-0.33263800	-0.36266400
C	1.24050200	-0.23724600	0.04988200
N	1.69698300	0.91421800	0.73257400
N	2.42271000	-0.80264400	-0.45795200
C	0.85854400	1.62971400	1.66991100
H	0.01409500	2.12082600	1.16341100
H	0.44029000	0.93638000	2.42095900
H	1.46049000	2.39523400	2.17626900
C	2.42390000	-2.01773700	-1.23861600
H	1.52043600	-2.58282200	-0.96069300
H	2.38355600	-1.81483500	-2.32271000
H	3.32513800	-2.60700000	-1.01630800
C	-3.09221800	0.97720400	-0.69141300
C	-3.53177600	-0.06707500	0.05859800
H	-3.64588000	1.77541300	-1.17276200
H	-4.53780700	-0.33268700	0.36264500
C	-1.24054500	-0.23740200	-0.05018600
N	-1.69704500	0.91407000	-0.73277400
C	-0.85846600	1.63015800	-1.66950800
H	-0.01533200	2.12293800	-1.16239500
H	-0.43827900	0.93681700	-2.41944800
H	-1.46091300	2.39436400	-2.17725000
C	-2.42384400	-2.01767600	1.23869500
H	-2.38323200	-1.81438800	2.32271400

H	-3.32517500	-2.60696000	1.01681500
H	-1.52049200	-2.58294100	0.96078400
N	-2.42275600	-0.80287700	0.45760100

Dimers

$C(NHC_{Me})_2$-Cl_2

Cl	-1.87392000	0.00245100	0.00066300
Cl	-4.46459700	0.00438900	-0.00030000
C	0.00342000	0.00032200	0.00071300
C	2.04441200	-2.89140900	0.82187000
C	1.05034100	-3.46779100	0.09643300
H	2.89902600	-3.33058500	1.32236500
H	0.87981000	-4.50722700	-0.15720900
C	0.64197100	-1.23784200	0.12370900
N	1.79919200	-1.52081900	0.84620900
N	0.19757600	-2.46478100	-0.34078400
C	2.46095300	-0.57215900	1.72307800
H	3.13421700	0.10471500	1.17479800
H	1.69897800	0.03639900	2.23322800
H	3.04427300	-1.12646700	2.46832400
C	-0.91865600	-2.67891200	-1.26068000
H	-1.88525400	-2.60334800	-0.74639200
H	-0.89725300	-1.91558400	-2.04912600
H	-0.79924400	-3.67361400	-1.70828300
C	2.05173900	2.88626300	-0.82272000
C	1.05944100	3.46551500	-0.09716500
H	2.90750900	3.32288200	-1.32346500
H	0.89192100	4.50549000	0.15629700
C	0.64545600	1.23658000	-0.12302300
N	1.80317700	1.51617200	-0.84597800
C	2.46142800	0.56564900	-1.72354500
H	3.13171000	-0.11443700	-1.17562800
H	1.69727500	-0.03935800	-2.23469500
H	3.04731400	1.11836100	-2.46794700
C	-0.91150900	2.68247400	1.26048500
H	-0.89285300	1.91921800	2.04902800
H	-0.78948400	3.67698500	1.70782000
H	-1.87804500	2.60967900	0.74554000
N	0.20447000	2.46492400	0.34116100

$C(NHC_{Me})_2$-Br_2

Br	-1.36934500	0.00058200	0.00011200
Br	-4.11799500	0.00037800	0.00002700
C	0.73953400	0.00013200	0.00002100
C	2.80375300	-2.85583300	0.88566100
C	1.85084900	-3.45280500	0.12285000

H	3.64804000	-3.27747000	1.41793300
H	1.71176800	-4.49536200	-0.13703300
C	1.38853300	-1.22803600	0.13973900
N	2.52704300	-1.49227100	0.90183600
N	0.99527000	-2.46581800	-0.34550700
C	3.15930900	-0.52387100	1.77783100
H	3.84367300	0.14589500	1.23430300
H	2.37978600	0.08932700	2.25434200
H	3.72495300	-1.05987900	2.54978900
C	-0.06940700	-2.70103200	-1.31605200
H	-1.05136100	-2.76545400	-0.82968800
H	-0.10204800	-1.86762800	-2.02879500
H	0.15362900	-3.63516200	-1.84784300
C	2.80665400	2.85406500	-0.88526000
C	1.85368700	3.45213500	-0.12343600
H	3.65173200	3.27477700	-1.41700900
H	1.71527400	4.49491300	0.13591300
C	1.38952300	1.22772200	-0.13969700
N	2.52891700	1.49067300	-0.90098700
C	3.16052800	0.52163000	-1.77675700
H	3.84217900	-0.15035800	-1.23258600
H	2.38056400	-0.08922400	-2.25559600
H	3.72906400	1.05712000	-2.54693800
C	-0.06813300	2.70261400	1.31453900
H	-0.10089600	1.87039300	2.02866800
H	0.15456900	3.63765700	1.84484800
H	-1.04993200	2.76611100	0.82780000
N	0.99690000	2.46604900	0.34471000

$C(NHC_{Me})_2$-I_2

I	-0.99218900	0.00009000	0.00002700
I	-4.01425000	0.00002900	-0.00000100
C	1.36984900	0.00010900	0.00012000
C	3.46137000	-2.81914200	0.94733800
C	2.54962300	-3.43579500	0.15112900
H	4.29377700	-3.22381100	1.51074800
H	2.44170600	-4.48082200	-0.11358300
C	2.03573300	-1.21735700	0.15482000
N	3.15528100	-1.46250900	0.95353300
N	1.69100100	-2.46487800	-0.34647200
C	3.75190700	-0.47816000	1.83608800
H	4.44125000	0.19505300	1.30321600
H	2.95203800	0.12865400	2.28608500
H	4.30446400	-0.99914300	2.62772200
C	0.68333500	-2.71894900	-1.36868400
H	-0.30529900	-2.90017000	-0.92608400

H	0.60338000	-1.84013600	-2.02048800
H	0.99864500	-3.59106300	-1.95715400
C	3.46244900	2.81858200	-0.94702300
C	2.55031900	3.43567000	-0.15161400
H	4.29530300	3.22288600	-1.51003400
H	2.44242800	4.48081200	0.11265700
C	2.03603600	1.21730000	-0.15480000
N	3.15617300	1.46195500	-0.95287900
C	3.75283700	0.47743400	-1.83521300
H	4.44164400	-0.19611800	-1.30207900
H	2.95299900	-0.12901400	-2.28577200
H	4.30602400	0.99826200	-2.62650900
C	0.68335500	2.71971000	1.36775300
H	0.60326100	1.84124300	2.02000100
H	0.99854500	3.59217600	1.95576000
H	-0.30519700	2.90084600	0.92494700
N	1.69117600	2.46509100	0.34577800

$C(NHC_{Me})_2$-ClF

Cl	0.00000500	2.20255500	0.00013100
F	0.00035800	4.24613800	0.00014300
C	0.00005700	0.30230500	0.00001100
C	-2.88024500	-1.76131500	0.81912300
C	-3.46239300	-0.76354200	0.10377400
H	-3.31536700	-2.61904600	1.31793000
H	-4.50341600	-0.59532800	-0.14509300
C	-1.23076500	-0.34754200	0.12236200
N	-1.50922300	-1.51388500	0.83645100
N	-2.46426400	0.09700000	-0.32922400
C	-0.55822900	-2.17273200	1.71179500
H	0.12046800	-2.84467800	1.16373600
H	0.05037100	-1.40834900	2.21897600
H	-1.10931900	-2.75675800	2.45921200
C	-2.68162000	1.20716200	-1.25456200
H	-2.57525200	2.17570600	-0.75114300
H	-1.93564300	1.16627900	-2.05937700
H	-3.68805800	1.09945400	-1.67907400
C	2.87993700	-1.76196000	-0.81896000
C	3.46233600	-0.76405900	-0.10400200
H	3.31479600	-2.62015300	-1.31720600
H	4.50342700	-0.59596700	0.14466400
C	1.23076300	-0.34782700	-0.12220300
N	1.50889300	-1.51447100	-0.83596200
C	0.55777400	-2.17277200	-1.71164400
H	-0.12172300	-2.84401000	-1.16372500
H	-0.04995400	-1.40802100	-2.21932300

H	1.10877500	-2.75747600	-2.45859100
C	2.68211300	1.20732700	1.25374000
H	1.93662300	1.16669400	2.05901200
H	3.68878300	1.10004300	1.67780800
H	2.57523400	2.17557500	0.74986400
N	2.46439100	0.09665400	0.32905200

$C(NHC_{Me})_2$-BrF

Br	2.12474600	-0.00108700	-0.00000900
F	4.19218700	-0.00220500	0.00000400
C	0.00838800	-0.00000800	0.00001200
C	-2.08901900	2.84033600	0.87694700
C	-1.12737200	3.44624600	0.13293800
H	-2.94110900	3.25515800	1.40216400
H	-0.98981000	4.49111100	-0.11855300
C	-0.65542300	1.22081400	0.13823900
N	-1.80908300	1.47641500	0.88409300
N	-0.25980600	2.46640900	-0.32838600
C	-2.44104300	0.50467800	1.75571500
H	-3.12068200	-0.16790100	1.20934500
H	-1.66061100	-0.10726700	2.23298700
H	-3.01155100	1.03683500	2.52708600
C	0.80801800	2.71040600	-1.29257300
H	1.78716100	2.77404900	-0.80137100
H	0.84809200	1.87875300	-2.00706000
H	0.58323700	3.64582500	-1.82222500
C	-2.09200500	-2.83817400	-0.87688900
C	-1.13098100	-3.44507500	-0.13288600
H	-2.94455900	-3.25211300	-1.40205200
H	-0.99450600	-4.49007800	0.11863000
C	-0.65668200	-1.22013800	-0.13824900
N	-1.81062000	-1.47454500	-0.88410900
C	-2.44147500	-0.50220000	-1.75585100
H	-3.12130200	0.17044700	-1.20978300
H	-1.66034900	0.10959700	-2.23216300
H	-3.01152600	-1.03378000	-2.52796300
C	0.80513200	-2.71120900	1.29264800
H	0.84614400	-1.87948300	2.00700100
H	0.57930800	-3.64627800	1.82247200
H	1.78423700	-2.77603400	0.80152500
N	-0.26237400	-2.46613800	0.32839200

$C(NHC_{Me})_2$-IF

I	-2.07249700	-0.00027900	-0.00000500
F	-4.20288600	-0.00045300	0.00000800
C	0.27213400	-0.00001800	0.00006000

C	2.41125900	-2.79331500	0.93812400
C	1.48948600	-3.42500900	0.16623100
H	3.25593200	-3.18556800	1.49200100
H	1.38590400	-4.47341400	-0.08678400
C	0.95685200	-1.20929300	0.15508100
N	2.09577300	-1.43785700	0.93357300
N	0.61275400	-2.46659300	-0.32375900
C	2.69234800	-0.44625800	1.80746500
H	3.37674000	0.22743200	1.26845400
H	1.89160300	0.16062900	2.25617700
H	3.25002500	-0.96010100	2.60048600
C	-0.40224100	-2.73857200	-1.33398600
H	-1.38245000	-2.93270000	-0.87903600
H	-0.50518500	-1.86110800	-1.98406200
H	-0.07885500	-3.60641500	-1.92519200
C	2.41011200	2.79408700	-0.93819700
C	1.48813000	3.42545100	-0.16628900
H	3.25455800	3.18666800	-1.49218700
H	1.38420400	4.47380400	0.08679700
C	0.95640000	1.20951300	-0.15499600
N	2.09522200	1.43846700	-0.93352700
C	2.69165400	0.44720300	-1.80793500
H	3.37674100	-0.22621300	-1.26946400
H	1.89084700	-0.16000800	-2.25610300
H	3.24854100	0.96134900	-2.60131400
C	-0.40293100	2.73809500	1.33443100
H	-0.50525600	1.86040300	1.98430800
H	-0.07973300	3.60590600	1.92578600
H	-1.38336900	2.93186000	0.87985900
N	0.61179600	2.46670300	0.32378700

$C(NHC_{Me})_2$-BrCl

Cl	-4.42712800	-0.00201300	0.00007400
Br	-1.84682600	-0.00138500	-0.00015700
C	0.26060700	-0.00015000	0.00004400
C	2.33730600	-2.84984800	0.88143300
C	1.38109500	-3.45034900	0.12576900
H	3.18490700	-3.26864300	1.41067500
H	1.24265200	-4.49375700	-0.13104900
C	0.91481400	-1.22596000	0.13897900
N	2.05880700	-1.48630900	0.89481300
N	0.52068200	-2.46620700	-0.33983200
C	2.69085200	-0.51635500	1.76911900
H	3.37036100	0.15684900	1.22369900
H	1.91092200	0.09363100	2.24927600
H	3.26173500	-1.05086300	2.53830700

C	-0.54613400	-2.70542500	-1.30713700
H	-1.52761100	-2.76213600	-0.81914600
H	-0.57713100	-1.87738400	-2.02626500
H	-0.32654500	-3.64439700	-1.83191400
C	2.33217500	2.85322700	-0.88156800
C	1.37527700	3.45192600	-0.12533600
H	3.17897100	3.27357300	-1.41087000
H	1.23521800	4.49505600	0.13174400
C	0.91263800	1.22680000	-0.13898000
N	2.05585500	1.48926100	-0.89526600
C	2.68931100	0.52054300	-1.76992300
H	3.37240100	-0.14971700	-1.22532400
H	1.91029200	-0.09260200	-2.24748300
H	3.25651800	1.05612200	-2.54109600
C	-0.55015200	2.70368600	1.30808900
H	-0.57978700	1.87525300	2.02683200
H	-0.33146600	3.64264800	1.83326900
H	-1.53195400	2.75939500	0.82064900
N	0.51663600	2.46627100	0.34028400

$C(NHC_{Me})_2$-ICl

Cl	-4.47086600	0.00008100	-0.00006700
I	-1.82600000	0.00035600	0.00004000
C	0.51320700	0.00006100	0.00010100
C	2.63131500	-2.80469500	0.94137100
C	1.71323000	-3.43010700	0.15975600
H	3.47135800	-3.20198100	1.49863900
H	1.60740100	-4.47709700	-0.09799000
C	1.18882900	-1.21407100	0.15551100
N	2.31921300	-1.44893600	0.94216500
N	0.84373300	-2.46647500	-0.33295200
C	2.91663200	-0.46027200	1.81940300
H	3.60347900	0.21240000	1.28253200
H	2.11675300	0.14698300	2.26884400
H	3.47192800	-0.97734900	2.61177900
C	-0.16918400	-2.73156600	-1.34766200
H	-0.24951900	-1.86188900	-2.01152900
H	0.14194300	-3.61289500	-1.92464100
H	-1.15640000	-2.90237200	-0.89834200
C	2.63226700	2.80403500	-0.94144500
C	1.71434300	3.42975800	-0.15987500
H	3.47256800	3.20102600	-1.49853500
H	1.60881200	4.47680400	0.09776700
C	1.18943100	1.21384400	-0.15542100
N	2.31991200	1.44833000	-0.94201200
C	2.91693700	0.45940700	-1.81923200

H	3.60202100	-0.21472200	-1.28195800
H	2.11678700	-0.14630400	-2.27030000
H	3.47411000	0.97626700	-2.61042200
C	-0.16849000	2.73165100	1.34736000
H	-0.24915400	1.86201700	2.01123200
H	0.14250300	3.61302500	1.92432900
H	-1.15546600	2.90246100	0.89750100
N	0.84464100	2.46637800	0.33293000

$C(NHC_{Me})_2$-ClNC

Cl	0.97606500	-1.71544400	0.09607800
N	3.47449300	-3.06087600	0.08897600
C	4.09119000	-2.09324500	-0.19152800
C	-0.06011600	-0.27610900	0.02097800
C	1.24143200	3.01447300	0.85732000
C	2.24567600	2.52481100	0.07755900
H	1.14716600	3.95851100	1.38027600
H	3.19631600	2.96275600	-0.20176000
C	0.63342800	0.95719800	0.13695000
N	0.24870600	2.04585000	0.90268700
N	1.86640600	1.27597100	-0.38123500
C	-0.89550400	2.06117900	1.79844600
H	-1.83271500	2.29122800	1.26909700
H	-0.99328200	1.07238700	2.26923300
H	-0.72663700	2.81895500	2.57266300
C	2.66304400	0.46350800	-1.30107000
H	3.25769900	-0.34217000	-0.78027100
H	1.99461600	-0.01918700	-2.02479900
H	3.35057500	1.13853700	-1.82593100
C	-3.57829200	-0.02241600	-0.84317000
C	-3.56447500	-1.20072100	-0.16977400
H	-4.39147500	0.50421400	-1.32781300
H	-4.36795400	-1.89380300	0.04885300
C	-1.44451600	-0.39893400	-0.14943600
N	-2.27606100	0.47618200	-0.84250100
C	-1.80233600	1.55122300	-1.69746100
H	-1.56394200	2.46317600	-1.12925000
H	-0.89333000	1.22133700	-2.22298900
H	-2.58173500	1.78527300	-2.43262300
C	-1.88617400	-2.51102300	1.17597300
H	-2.80574000	-2.98448300	1.54076200
H	-1.25420800	-3.25467200	0.67494400
H	-1.32436600	-2.10219500	2.02665200
N	-2.26558100	-1.43384000	0.26649100

$C(NHC_{Me})_2$-BrNC

N	-4.24351600	-0.00106500	-0.00017100
C	-5.42622400	-0.00108800	-0.00004700
Br	-1.91460900	-0.00065100	-0.00007200
C	0.14715800	-0.00008900	0.00012800
C	2.25134500	-2.83271300	0.87966700
C	1.29429500	-3.44404600	0.13415000
H	3.10675200	-3.24194000	1.40370300
H	1.16203900	-4.48943600	-0.11776300
C	0.81070000	-1.22523000	0.14343200
N	1.96043400	-1.47175600	0.89168000
N	0.42221800	-2.46852900	-0.32803700
C	2.58981000	-0.49665900	1.76357400
H	3.26870600	0.17534300	1.21625100
H	1.80805100	0.11276100	2.24090100
H	3.16041400	-1.02762600	2.53524200
C	-0.64730400	-2.72187300	-1.28974200
H	-1.62576000	-2.78445600	-0.79670300
H	-0.68702600	-1.90005300	-2.01537400
H	-0.42303900	-3.66275700	-1.80851600
C	2.24839700	2.83444400	-0.88035400
C	1.29125600	3.44486500	-0.13417400
H	3.10302400	3.24448000	-1.40502800
H	1.15815500	4.49014200	0.11776400
C	0.80977700	1.22561600	-0.14326500
N	1.95866800	1.47325500	-0.89231300
C	2.58861000	0.49861900	-1.76431700
H	3.26648700	-0.17415400	-1.21670600
H	1.80722900	-0.11007300	-2.24321400
H	3.16044900	1.03005300	-2.53472900
C	-0.64836700	2.72066400	1.29135500
H	-0.68671800	1.89839400	2.01657300
H	-0.42421800	3.66141200	1.81041700
H	-1.62746100	2.78273900	0.79950600
N	0.42041400	2.46850900	0.32851900

$C(NHC_{Me})_2$-INC

I	-1.90459300	0.00059700	0.00034000
N	-4.23549100	0.00096800	-0.00018300
C	-5.41810300	-0.00358400	-0.00183900
C	0.41213900	-0.00032700	0.00056900
C	2.55245500	-2.79127100	0.94024700
C	1.63350700	-3.42491500	0.16666500
H	3.39822300	-3.18134900	1.49388600
H	1.53199900	-4.47334000	-0.08690700
C	1.09635400	-1.21250500	0.15853500

N	2.23188100	-1.43744300	0.93869000
N	0.75531300	-2.46767300	-0.32359600
C	2.82823900	-0.44442100	1.81264800
H	3.50921400	0.23086300	1.27186900
H	2.02748800	0.15878700	2.26565200
H	3.38962400	-0.95821000	2.60271900
C	-0.25959900	-2.74272100	-1.33406000
H	-0.34893900	-1.87551300	-1.99995200
H	0.05594700	-3.62245300	-1.91068100
H	-1.24322200	-2.92271300	-0.88056000
C	2.55008700	2.79136600	-0.94232900
C	1.63260300	3.42449300	-0.16659400
H	3.39469100	3.18176900	-1.49751100
H	1.53143300	4.47281000	0.08757800
C	1.09581000	1.21201500	-0.15839200
N	2.22961500	1.43751100	-0.94078900
C	2.82488300	0.44482200	-1.81585300
H	3.50925100	-0.22845800	-1.27684800
H	2.02365000	-0.16048700	-2.26514600
H	3.38219700	0.95887400	-2.60864600
C	-0.25675800	2.74171200	1.33817300
H	-0.34755900	1.87242500	2.00110700
H	0.06270500	3.61837300	1.91739100
H	-1.24079200	2.92693100	0.88757500
N	0.75551600	2.46693200	0.32500000

$C(NHC_{Me})_2$-HOCl

O	-0.00509100	4.34478500	-0.07379000
H	-0.36470800	4.50982700	0.81846500
Cl	-0.00539800	2.29655800	-0.01386400
C	0.00280900	0.25189200	0.00971100
C	-2.90681100	-1.75617000	0.84795600
C	-3.46692000	-0.78455000	0.08031600
H	-3.35988400	-2.58949300	1.37189300
H	-4.50182900	-0.61948000	-0.19505100
C	-1.22600200	-0.38265500	0.12505200
N	-1.53416500	-1.52212800	0.87745600
N	-2.45175000	0.04735300	-0.37053800
C	-0.59206300	-2.17463700	1.76407000
H	0.07952600	-2.86376400	1.22795800
H	0.02629000	-1.40778800	2.25618500
H	-1.14794600	-2.73979000	2.52272000
C	-2.63471600	1.12352900	-1.33786200
H	-2.63865900	2.10767100	-0.85259700
H	-1.80179800	1.11367800	-2.05273000
H	-3.58151900	0.95161400	-1.86718100

C	2.91327200	-1.74908500	-0.84324200
C	3.47771200	-0.76509000	-0.09517800
H	3.36420600	-2.58569500	-1.36372100
H	4.51521200	-0.58952500	0.16329100
C	1.23405000	-0.37609600	-0.11559500
N	1.53894800	-1.52302200	-0.85781100
C	0.59237900	-2.18825800	-1.73053800
H	-0.08104300	-2.86421400	-1.18036600
H	-0.02315200	-1.42951400	-2.23830500
H	1.14550900	-2.77035500	-2.47821200
C	2.65523600	1.16521700	1.29727000
H	1.84433700	1.15426800	2.03742400
H	3.61991600	1.02037900	1.80173900
H	2.62835200	2.13793000	0.78989200
N	2.46353900	0.06635500	0.35844300

$C(NHC_{Me})_2$-HOBr

O	-4.29454000	0.00393800	-0.07302700
H	-4.48961100	-0.34040700	0.81891100
Br	-2.20005900	-0.00443700	-0.00987200
C	0.04956200	0.00163600	0.00930000
C	2.10682400	-2.86346200	0.89112100
C	1.16096100	-3.45103900	0.11261600
H	2.94149400	-3.29375800	1.43194200
H	1.02355600	-4.49117100	-0.15815800
C	0.70309500	-1.21703800	0.13877200
N	1.84010200	-1.49707300	0.90774300
N	0.31393300	-2.45675200	-0.35771000
C	2.46800500	-0.53436600	1.78990600
H	3.15398500	0.13990500	1.25308900
H	1.68600600	0.07859100	2.26419700
H	3.03147800	-1.07217900	2.56281800
C	-0.73261300	-2.67219200	-1.34817200
H	-1.71336100	-2.81754500	-0.87662000
H	-0.80438900	-1.78626400	-1.99115300
H	-0.46592800	-3.55172000	-1.95043600
C	2.09108100	2.87305400	-0.88932400
C	1.13798800	3.46091200	-0.12016600
H	2.92463800	3.30423900	-1.43110000
H	0.99121700	4.50248500	0.13981500
C	0.69517500	1.22373200	-0.12898000
N	1.83345300	1.50474400	-0.89545300
C	2.46878800	0.54107600	-1.77157500
H	3.15153300	-0.13152800	-1.22870300
H	1.69161200	-0.07301200	-2.25206800
H	3.03749100	1.07882900	-2.54062100

C	-0.76487000	2.68340700	1.32882000
H	-0.82235500	1.81393500	1.99571800
H	-0.52327400	3.58461800	1.90905200
H	-1.74309200	2.79387600	0.84260600
N	0.29585500	2.46487700	0.35492700

$C(NHC_{Me})_2$-HOI

O	-4.30529300	0.00447100	-0.07630300
H	-4.54937000	-0.32529200	0.80831900
I	-2.12400500	-0.00856300	-0.00681300
C	0.32891400	-0.00054300	0.00865300
C	2.45064300	-2.80694900	0.93967400
C	1.53560300	-3.42766300	0.15089700
H	3.28708800	-3.20878400	1.49936500
H	1.43202600	-4.47328600	-0.11361400
C	1.01003600	-1.20583800	0.15427200
N	2.14331600	-1.44947700	0.94172000
N	0.66825800	-2.46068200	-0.34045500
C	2.73864500	-0.46330400	1.82071900
H	3.41893100	0.21743300	1.28497700
H	1.93736200	0.13869100	2.27596100
H	3.30115200	-0.98023500	2.60853700
C	-0.33783200	-2.71487300	-1.36174800
H	-1.31260300	-2.95968200	-0.91877300
H	-0.46701800	-1.80883300	-1.96643600
H	0.00750300	-3.54325700	-1.99648000
C	2.40266000	2.83391600	-0.94556500
C	1.48564300	3.44323800	-0.15026500
H	3.22779700	3.24650600	-1.51411200
H	1.36819700	4.48798900	0.11180600
C	0.99200500	1.21431600	-0.14497300
N	2.11462400	1.47224600	-0.94230200
C	2.71492400	0.49352100	-1.82660800
H	3.40727700	-0.17922600	-1.29634500
H	1.91751800	-0.11728700	-2.27665600
H	3.26518900	1.01783500	-2.61812200
C	-0.37075100	2.70722500	1.37251900
H	-0.03655200	3.54261800	2.00404400
H	-1.34946000	2.93592900	0.92938800
H	-0.48452300	1.80051800	1.97944400
N	0.63682500	2.46479200	0.35029100

$C(NHC_{Me})_2$-ClCN

C	-3.91314000	1.75640900	0.23379000
N	-5.00969300	2.13099900	0.39429600
Cl	-2.34458000	1.22456000	0.00492200

C	0.23563300	0.12633500	-0.32520700
C	-0.53950600	-3.17418700	0.98957300
C	-1.25464800	-3.16851400	-0.16613700
H	-0.47486000	-3.92204600	1.77146100
H	-1.91578100	-3.91917000	-0.58323300
C	-0.11227600	-1.18137100	-0.07278700
N	0.17408200	-1.97794600	1.04781300
N	-1.00078400	-1.96704800	-0.81522300
C	0.87705800	-1.46990700	2.20801100
H	1.96612500	-1.42736900	2.04856900
H	0.52684800	-0.44768800	2.42335700
H	0.66878600	-2.11930400	3.06770800
C	-1.49968800	-1.58358500	-2.12033700
H	-1.19799800	-0.53973500	-2.28416000
H	-1.06621800	-2.21350700	-2.91438700
H	-2.59615000	-1.66098100	-2.15632500
C	3.73503900	1.11387200	-0.23061100
C	3.10833600	2.29847100	-0.01465600
H	4.79083500	0.88481000	-0.31882300
H	3.51839700	3.29096300	0.13166400
C	1.47115700	0.69558000	-0.16014200
N	2.75468100	0.12040000	-0.29105000
C	2.96549800	-1.21327900	-0.81311600
H	2.68998200	-1.99235100	-0.08582200
H	2.35430500	-1.36518200	-1.71901000
H	4.02605100	-1.33166800	-1.06910700
C	0.70267500	3.04952200	0.18674800
H	0.10112300	2.86545400	1.08994800
H	1.16901500	4.04027300	0.25848800
H	0.02554700	3.02325100	-0.68104500
N	1.74100200	2.05368800	0.04220400

$C(NHC_{Me})_2$-BrCN

Br	-2.18905600	-0.00163100	0.00002100
C	-4.17885100	-0.00244900	-0.00020700
N	-5.34955500	-0.00289300	-0.00028400
C	0.26496400	-0.00018800	0.00035200
C	2.30370000	-2.87676700	0.89486700
C	1.36124500	-3.45922500	0.10930800
H	3.13008100	-3.31294800	1.44380900
H	1.21875600	-4.49875200	-0.16118400
C	0.91551800	-1.21636400	0.13281600
N	2.04832900	-1.50792200	0.90650300
N	0.52515900	-2.45711300	-0.36730600
C	2.68065900	-0.54823300	1.78864900
H	3.35899600	0.13141000	1.24911500

H	1.90198300	0.05942600	2.27526400
H	3.25385100	-1.08829200	2.55274500
C	-0.52832900	-2.66178100	-1.34768800
H	-0.66994400	-1.72971700	-1.90888500
H	-0.22899600	-3.46654900	-2.03364400
H	-1.48412300	-2.92133900	-0.87065300
C	2.29682200	2.88071700	-0.89589500
C	1.35406400	3.46109000	-0.10914900
H	3.12160900	3.31870900	-1.44579100
H	1.20975200	4.50027200	0.16170800
C	0.91294000	1.21732600	-0.13259800
N	2.04424100	1.51135400	-0.90751600
C	2.67758200	0.55308900	-1.79048800
H	3.35820900	-0.12496800	-1.25184100
H	1.89961800	-0.05637200	-2.27597800
H	3.24847500	1.09442000	-2.55540500
C	-0.53191100	2.65933600	1.35020300
H	-0.67182200	1.72624500	1.91010200
H	-0.23258100	3.46337300	2.03703700
H	-1.48852100	2.91866000	0.87469100
N	0.52057300	2.45717500	0.36819700

$C(NHC_{Me})_2$-ICN

I	-2.01244500	0.00053200	0.00000900
C	-4.23557300	0.00079600	0.00007100
N	-5.40667900	0.00101600	0.00004500
C	0.49714100	0.00009400	-0.00032900
C	2.59052300	-2.82596800	0.93926100
C	1.67272600	-3.43834400	0.14770300
H	3.42053100	-3.23582300	1.50268500
H	1.55878200	-4.48309400	-0.11586600
C	1.16939600	-1.21015900	0.14962500
N	2.29691200	-1.46543100	0.93967000
N	0.81691400	-2.46160200	-0.34621300
C	2.90071500	-0.48508700	1.82041900
H	3.58272000	0.19317700	1.28416700
H	2.10495100	0.11930100	2.28182600
H	3.46324800	-1.00801000	2.60397700
C	-0.19440900	-2.70452600	-1.36389400
H	-0.33125900	-1.78798900	-1.95132000
H	0.14888700	-3.51731500	-2.01892400
H	-1.16328200	-2.97110200	-0.91937600
C	2.59377300	2.82418900	-0.93850800
C	1.67594100	3.43751600	-0.14775200
H	3.42453400	3.23321400	-1.50142800
H	1.56269700	4.48238700	0.11563000

C	1.17048000	1.20979000	-0.14986200
N	2.29889600	1.46393100	-0.93902200
C	2.90251900	0.48297600	-1.81920600
H	3.58279900	-0.19641600	-1.28220400
H	2.10658400	-0.12016900	-2.28196200
H	3.46693300	1.00535000	-2.60177400
C	-0.19319600	2.70558000	1.36235500
H	-0.33019000	1.78979800	1.95092600
H	0.14952900	3.51930200	2.01651900
H	-1.16187000	2.97140000	0.91694100
N	0.81878300	2.46160700	0.34557800

$C(NHC_{Me})_2$-NH_2Cl

N	-2.28376700	3.94943600	0.00608000
H	-2.22823300	4.20606600	1.00230600
H	-1.63463400	4.60489000	-0.45258700
Cl	-1.21660200	2.33825500	-0.00103700
C	0.00185100	0.17802800	-0.06794600
C	-1.98861300	-2.68454500	0.94889600
C	-2.81576000	-2.09482100	0.04591400
H	-2.13595100	-3.55962200	1.57122900
H	-3.81576800	-2.36847400	-0.26958000
C	-0.88916600	-0.86012800	0.08823100
N	-0.80577300	-1.94958800	0.97205500
N	-2.15329500	-0.99813200	-0.48843100
C	0.26082300	-2.10022400	1.93847900
H	1.17194800	-2.53002800	1.49203400
H	0.51625000	-1.10908200	2.34610700
H	-0.07907800	-2.75585100	2.75054800
C	-2.65592300	-0.17146400	-1.57230200
H	-1.80834800	0.37695200	-2.00075800
H	-3.11800300	-0.81219100	-2.33864400
H	-3.38662300	0.56619900	-1.21206400
C	3.48848900	-0.57110700	-0.76328500
C	3.60197900	0.60598100	-0.09627400
H	4.24589700	-1.19387700	-1.22534500
H	4.48126700	1.19051600	0.14835000
C	1.36991500	0.08458000	-0.14712700
N	2.13615700	-0.90827900	-0.79049700
C	1.55205600	-1.95756400	-1.59732200
H	1.15151800	-2.78263700	-0.98660000
H	0.72186400	-1.54076200	-2.19007500
H	2.31831500	-2.35862200	-2.27331900
C	2.04604300	2.12530600	1.16606600
H	2.19407500	1.83934000	2.22204600
H	2.71578100	2.96312900	0.92615700

H	1.00384700	2.43638900	1.02019700
N	2.32638300	1.01414500	0.27617100

$C(NHC_{Me})_2$-NH_2Br

N	4.36272700	-0.90552700	-0.03164600
H	4.52487500	-1.14952500	0.95674200
H	4.39427900	-1.81796000	-0.51038700
Br	2.29503500	-0.57121600	-0.00119900
C	-0.14258300	-0.02051600	-0.03341800
C	-1.18077300	3.31876400	0.95386200
C	-0.16553300	3.60435400	0.09720100
H	-1.80135600	3.97453000	1.55343200
H	0.25651900	4.55871800	-0.19491100
C	-0.39737000	1.32903300	0.11757900
N	-1.33969300	1.93553900	0.96394100
N	0.30434000	2.40612000	-0.42402400
C	-2.16577000	1.19268900	1.89201600
H	-3.05002600	0.75224800	1.40420700
H	-1.56961000	0.37314000	2.32375400
H	-2.50028000	1.86508300	2.69240000
C	1.29284200	2.30164700	-1.48502100
H	1.15680000	1.33752000	-1.99011300
H	1.14351800	3.12493900	-2.19917400
H	2.31736200	2.33024700	-1.08897000
C	-2.95054900	-2.17671200	-0.85562400
C	-2.17994300	-3.02375900	-0.12659000
H	-3.88726600	-2.34622600	-1.37396600
H	-2.33093900	-4.06655800	0.12704800
C	-1.10336200	-1.00287900	-0.15110200
N	-2.31140600	-0.93875300	-0.86985200
C	-2.67578200	0.17905600	-1.71409000
H	-3.09102900	1.02140100	-1.13795800
H	-1.77948300	0.53802200	-2.24434000
H	-3.42455400	-0.15309300	-2.44471300
C	-0.12359500	-2.82025300	1.29719300
H	-0.50292100	-2.64458600	2.31879300
H	0.02038600	-3.89927000	1.14967400
H	0.83755600	-2.30492500	1.17573900
N	-1.05675900	-2.32447600	0.30053500

$C(NHC_{Me})_2$-NH_2I

N	4.44958300	-0.18236300	-0.04371100
H	4.68362000	-0.39860800	0.93618400
H	4.65352700	-1.06249300	-0.53919600
I	2.21097600	-0.17898000	-0.00075100
C	-0.40694600	-0.01428500	-0.02623500

C	-2.07002500	3.05566400	0.99106400
C	-1.13278400	3.54275700	0.13701100
H	-2.80369200	3.57114100	1.59984000
H	-0.90510800	4.56384200	-0.14533600
C	-0.91377200	1.26596000	0.13623400
N	-1.95373100	1.66856600	0.98844800
N	-0.43885700	2.46398600	-0.39616200
C	-2.62294400	0.77183500	1.90749200
H	-3.41104600	0.18091900	1.41433700
H	-1.88165200	0.07354900	2.32696400
H	-3.07377300	1.35950500	2.71739500
C	0.52442300	2.56917900	-1.48036300
H	0.57418800	1.60164100	-1.99499800
H	0.19399100	3.35033600	-2.18093300
H	1.53122000	2.80281700	-1.10734500
C	-2.84057100	-2.56853600	-0.91103900
C	-1.97364500	-3.29289000	-0.15886100
H	-3.72664400	-2.87396300	-1.45543100
H	-1.97519900	-4.34666000	0.09417000
C	-1.20559400	-1.13658200	-0.16348800
N	-2.38884800	-1.25103600	-0.91402500
C	-2.89729900	-0.19907100	-1.76916500
H	-3.45948000	0.56294700	-1.20622400
H	-2.05108500	0.29878400	-2.26791400
H	-3.55893000	-0.64116200	-2.52515400
C	-0.03391400	-2.78712900	1.34349400
H	-0.46329900	-2.59878400	2.34264400
H	0.21520400	-3.85296700	1.25524700
H	0.88043200	-2.19070500	1.22789900
N	-0.97751100	-2.43566700	0.29556700

$C(NHC_{Me})_2$-$ClCF_3$

C	-3.81921900	0.67071500	0.14817600
F	-4.15114000	0.50278300	1.44620100
F	-4.47605200	-0.26313100	-0.57347800
F	-4.26294000	1.88535900	-0.24077800
Cl	-2.04840000	0.53096200	-0.08462200
C	0.87927700	0.06592500	-0.41353400
C	0.67929700	-3.27827600	0.99531200
C	0.08573000	-3.45545600	-0.21461500
H	0.82554000	-3.97379600	1.81371000
H	-0.36549900	-4.34002900	-0.64887700
C	0.78533300	-1.27141500	-0.12364900
N	1.12847900	-1.95915100	1.05453600
N	0.14455100	-2.24740400	-0.89694400
C	1.59338000	-1.27755100	2.24434700

H	2.65662500	-0.99740900	2.17493800
H	1.00958500	-0.35330700	2.38410600
H	1.45568500	-1.93454600	3.11265600
C	-0.32750200	-1.99783000	-2.24218900
H	-0.38792900	-0.90690000	-2.36404600
H	0.36687400	-2.40182000	-2.99826300
H	-1.32075500	-2.44585100	-2.38730200
C	4.01947500	1.86515600	-0.07739700
C	3.11221600	2.87167000	0.00883800
H	5.10298400	1.89481100	-0.05996900
H	3.26078400	3.93861300	0.12876900
C	1.92182200	0.91566300	-0.17269200
N	3.31308900	0.66158300	-0.15207200
C	3.87789500	-0.59878400	-0.58466900
H	3.67553400	-1.40866700	0.13249400
H	3.44872200	-0.89569000	-1.55750600
H	4.96457100	-0.48513200	-0.68837700
C	0.58656900	3.02324600	-0.05072300
H	0.01182500	2.84884700	0.87261900
H	0.78856700	4.09725900	-0.15457200
H	-0.02578600	2.67418300	-0.89602800
N	1.84259900	2.30777300	-0.03644900

$C(NHC_{Me})_2$-$BrCF_3$

C	-3.78767600	0.38822200	0.11730500
F	-4.16966200	0.20369700	1.40373700
F	-4.37693900	-0.57932300	-0.62729400
F	-4.28624500	1.57978300	-0.29097800
Br	-1.77649800	0.33191200	-0.06871200
C	0.97019000	0.07333200	-0.27085300
C	1.48834100	-3.28778900	1.01707700
C	0.77438300	-3.53992000	-0.11203600
H	1.85079900	-3.96173100	1.78464100
H	0.41114200	-4.47789700	-0.51543100
C	1.11946500	-1.27743800	-0.02821400
N	1.71471700	-1.91433900	1.06998600
N	0.55101600	-2.32817700	-0.75107300
C	2.24609800	-1.18907200	2.20605800
H	3.26899900	-0.82197400	2.02505700
H	1.60149000	-0.31864100	2.40389500
H	2.25468800	-1.84880700	3.08308600
C	-0.09492100	-2.15895200	-2.03873000
H	-0.10378000	-1.08309400	-2.25695200
H	0.46131000	-2.69111500	-2.82717500
H	-1.13018400	-2.52753900	-2.00923300
C	3.95541200	2.15170100	-0.32683400

C	2.99504500	3.06569200	-0.03695700
H	5.02146700	2.27640600	-0.47773600
H	3.07046100	4.13521100	0.12084200
C	1.95360100	1.02490300	-0.15647700
N	3.34243800	0.89795200	-0.37802400
C	3.94600900	-0.29006300	-0.94306500
H	4.03345100	-1.10534100	-0.20801500
H	3.33334900	-0.65607600	-1.78383300
H	4.94956900	-0.03920800	-1.30983300
C	0.50072300	3.00924800	0.34206900
H	0.00652700	2.52764800	1.19779700
H	0.66072000	4.07226500	0.56305400
H	-0.16665900	2.91105100	-0.52727300
N	1.78464100	2.39246700	0.08150500

$C(NHC_{Me})_2$-ICF_3

C	-3.89584500	-0.00553700	-0.00109000
F	-4.41196600	0.79968700	0.97436200
F	-4.40958800	-1.25354200	0.20964100
F	-4.41337800	0.43518700	-1.18509500
I	-1.60720100	-0.00490900	0.00000100
C	1.05102700	-0.00222900	0.00334900
C	3.12167900	-2.84630500	0.92961300
C	2.20231400	-3.44624900	0.13021100
H	3.94558900	-3.26747300	1.49387200
H	2.08205200	-4.48859000	-0.14027600
C	1.71548100	-1.20994000	0.14307400
N	2.84248300	-1.48236800	0.93436600
N	1.35637900	-2.45948200	-0.36167400
C	3.45064000	-0.50899300	1.81769100
H	4.12298300	0.17904800	1.28118900
H	2.65761100	0.08817700	2.29414900
H	4.02540000	-1.03630200	2.58966100
C	0.33772000	-2.68416700	-1.37332200
H	0.17338800	-1.74558600	-1.91745800
H	0.68567100	-3.46156200	-2.06864000
H	-0.61959600	-2.98904600	-0.92725600
C	3.07355900	2.86992300	-0.94223800
C	2.15952600	3.45562500	-0.12614800
H	3.88319600	3.30329800	-1.51780300
H	2.03095000	4.49541200	0.15030200
C	1.69949700	1.21388000	-0.14115700
N	2.81033500	1.50294300	-0.94865000
C	3.41781300	0.53927600	-1.84307300
H	4.10953600	-0.13872100	-1.31853300
H	2.62540400	-0.06951300	-2.30555700

H	3.97097900	1.07537600	-2.62469800
C	0.32978000	2.66416600	1.40491900
H	0.17386100	1.71583800	1.93434300
H	0.68786000	3.42925500	2.10885100
H	-0.63397600	2.97659200	0.97849400
N	1.33308600	2.45680900	0.37427100

$C(NHC_{Me})_2$-C_6F_5Cl

C	5.14284500	-0.22742100	0.11955500
C	4.41180600	-1.38199000	0.41278000
C	3.02012300	-1.37174500	0.29063400
C	2.33512900	-0.22307500	-0.12210900
C	3.08405000	0.92322500	-0.41150200
C	4.47609400	0.92913700	-0.29417200
F	2.34362500	-2.49879800	0.58043100
F	5.05250500	-2.49555000	0.81104200
F	6.48094600	-0.22934700	0.23499300
F	5.17895800	2.04068600	-0.57709500
F	2.47109800	2.05274600	-0.81290900
Cl	0.59176800	-0.22126600	-0.27147900
C	-2.36511500	-0.01132000	-0.42719100
C	-2.42819300	3.03295800	1.55618100
C	-1.91413300	3.48301400	0.38089900
H	-2.60416100	3.55363400	2.49044500
H	-1.57307500	4.47288900	0.10080800
C	-2.39111500	1.25696700	0.09574700
N	-2.74344300	1.68480200	1.38832700
N	-1.89087400	2.41688400	-0.50792000
C	-3.07933700	0.75790200	2.44886600
H	-4.10712800	0.37293600	2.35434800
H	-2.39218300	-0.10296400	2.41059900
H	-2.97877400	1.26647900	3.41619400
C	-1.44799000	2.45878900	-1.88537000
H	-1.33229700	1.41642600	-2.21494800
H	-2.18705200	2.95946900	-2.53302400
H	-0.48424000	2.98193100	-1.96246400
C	-5.29261200	-2.16274000	-0.31714100
C	-4.28778600	-3.06783000	-0.43890300
H	-6.36581400	-2.30850500	-0.27127100
H	-4.32468200	-4.14905600	-0.50512000
C	-3.30559400	-0.99625700	-0.30905800
N	-4.71199000	-0.89692800	-0.20110400
C	-5.41866600	0.35321200	-0.38178000
H	-5.26877300	1.03816700	0.46662800
H	-5.06302800	0.86370600	-1.29355100
H	-6.49176200	0.14473500	-0.47925600

C	-1.76568600	-2.94158800	-0.60334300
H	-1.16197100	-2.85775900	0.31360100
H	-1.86744200	-4.00008800	-0.87570100
H	-1.23639100	-2.39902700	-1.40137500
N	-3.08346300	-2.37425900	-0.42408700

$C(NHC_{Me})_2$-C_6F_5Br

C	5.21725500	-0.00021400	0.00014300
C	4.51636400	-1.19661200	0.17583700
C	3.11914500	-1.17893600	0.17304200
C	2.40167500	-0.00017700	-0.00015600
C	3.11921400	1.17854800	-0.17320900
C	4.51643600	1.19620000	-0.17570400
F	2.47660300	-2.36097300	0.34718100
F	5.19791200	-2.34903800	0.34551700
F	6.56348600	-0.00023600	0.00028600
F	5.19803800	2.34861200	-0.34524700
F	2.47672800	2.36060700	-0.34748900
Br	0.37222800	-0.00023700	-0.00023600
C	-2.18194800	-0.00015500	-0.00015500
C	-4.17896500	2.82508100	1.11664100
C	-3.23935100	3.45127100	0.36141000
H	-4.99414000	3.23088500	1.70438300
H	-3.08885200	4.50579500	0.16266400
C	-2.81787900	1.20602300	0.22174100
N	-3.94036400	1.45600000	1.02787900
N	-2.42094000	2.47663700	-0.19562900
C	-4.57623600	0.44043400	1.84076700
H	-5.26435700	-0.19176900	1.25736500
H	-3.80052200	-0.20939400	2.27547500
H	-5.13997100	0.92731000	2.64673400
C	-1.36738300	2.73530800	-1.16165400
H	-1.23798800	1.84001000	-1.78286900
H	-1.65822100	3.58752900	-1.79205900
H	-0.40697300	2.94905300	-0.67130700
C	-4.18194000	-2.82378000	-1.11570600
C	-3.24208300	-3.45076400	-0.36143900
H	-4.99801800	-3.22889100	-1.70267100
H	-3.09219600	-4.50542700	-0.16296600
C	-2.81893500	-1.20581800	-0.22170600
N	-3.94234900	-1.45486900	-1.02684200
C	-4.57811600	-0.43874600	-1.83911300
H	-5.26475100	0.19435600	-1.25494200
H	-3.80226900	0.21012800	-2.27502300
H	-5.14347500	-0.92516300	-2.64421800
C	-1.36792400	-2.73644700	1.15969100

H	-1.23747500	-1.84163700	1.78138600
H	-1.65828500	-3.58900500	1.78986200
H	-0.40815000	-2.95020000	0.66809500
N	-2.42252200	-2.47681400	0.19509600

$C(NHC_{Me})_2$-C_6F_5I

C	5.38558000	-0.00010600	-0.00012700
C	4.68461400	-1.18936800	0.21797100
C	3.28730200	-1.16675200	0.21399500
C	2.56403900	-0.00017800	0.00015600
C	3.28720200	1.16642100	-0.21381600
C	4.68451300	1.18912000	-0.21808200
F	2.64842800	-2.34860400	0.43130500
F	5.36877000	-2.33562900	0.42834400
F	6.73306400	-0.00007200	-0.00025400
F	5.36855600	2.33542300	-0.42857500
F	2.64821600	2.34825500	-0.43099500
I	0.28908000	-0.00036100	0.00005900
C	-2.27513300	-0.00012300	-0.00016800
C	-4.34128800	2.73962500	1.20939600
C	-3.42326300	3.41594400	0.47159600
H	-5.16480000	3.10204900	1.81347800
H	-3.30302800	4.47982300	0.30515000
C	-2.93721900	1.19260600	0.26306100
N	-4.05950300	1.38262800	1.08091400
N	-2.57828100	2.48328600	-0.11687300
C	-4.66692900	0.32700300	1.86587200
H	-5.35160400	-0.29573700	1.26886100
H	-3.87351000	-0.32179500	2.26769300
H	-5.22756600	0.77602800	2.69540300
C	-1.56532300	2.81013800	-1.10838100
H	-1.44278600	1.95258600	-1.78199100
H	-1.89796700	3.68761300	-1.68036800
H	-0.59121900	3.01659500	-0.64376700
C	-4.34401300	-2.73821400	-1.20884200
C	-3.42610800	-3.41528800	-0.47158500
H	-5.16832100	-3.10001600	-1.81221500
H	-3.30656200	-4.47927200	-0.30531900
C	-2.93820900	-1.19233500	-0.26315800
N	-4.06114300	-1.38144800	-1.08042700
C	-4.66815000	-0.32534700	-1.86505600
H	-5.35274500	0.29738700	-1.26793400
H	-3.87444400	0.32333600	-2.26648000
H	-5.22877200	-0.77386600	-2.69487900
C	-1.56648400	-2.81109200	1.10700900
H	-1.44255800	-1.95371000	1.78057500

H	-1.89936400	-3.68831300	1.67924700
H	-0.59301400	-3.01837500	0.64143700
N	-2.58008400	-2.48330800	0.11646300

$C(NHC_{Me})_2$-C_5H_4NCl

C	5.70488600	-1.37926500	-0.40469400
C	4.32099200	-1.41300800	-0.59851800
C	3.54921700	-0.42689100	0.02062200
C	4.18479900	0.54293500	0.79979100
C	5.57559600	0.47628400	0.92203000
N	6.34163000	-0.45986500	0.33869500
H	6.33632700	-2.13867000	-0.87728400
H	3.85664100	-2.18386800	-1.21420600
H	3.61157400	1.32669000	1.29569100
H	6.10227000	1.22223100	1.52615000
Cl	1.81547900	-0.40590100	-0.17516900
C	-1.51872200	-0.10026300	-0.49334500
C	-1.18341000	3.26345700	0.83599200
C	-0.71314300	3.41926500	-0.42968600
H	-1.25464400	3.97592800	1.64995100
H	-0.31505800	4.29837900	-0.92326300
C	-1.40102600	1.23531400	-0.23400800
N	-1.63432100	1.94818000	0.96087000
N	-0.84180400	2.20114400	-1.08458500
C	-1.94909000	1.28137900	2.20611700
H	-2.98229000	0.90025000	2.22042500
H	-1.27300900	0.42149100	2.34941000
H	-1.82214100	1.98989300	3.03471400
C	-0.47158200	1.91208300	-2.45161900
H	-0.31661400	0.82492400	-2.52098500
H	-1.26563000	2.19784400	-3.16273200
H	0.45480100	2.44319700	-2.71277200
C	-4.50985900	-2.02429600	0.24693800
C	-3.55193200	-2.98734800	0.24923100
H	-5.58195300	-2.10250900	0.38767700
H	-3.63663900	-4.05619700	0.40846900
C	-2.48418800	-0.98368100	-0.11170600
N	-3.87465700	-0.79266200	0.06752200
C	-4.53962500	0.43240000	-0.32108000
H	-4.26231800	1.27300400	0.33280000
H	-4.26482700	0.70704900	-1.35469700
H	-5.62543800	0.28348600	-0.26174200
C	-1.04117500	-3.02033300	-0.08384600
H	-0.46865000	-2.98762500	0.85837700
H	-1.18861700	-4.06777700	-0.38082100
H	-0.45922500	-2.48585600	-0.84998100

N	-2.32486500	-2.36962000	0.04026600

$C(NHC_{Me})_2$-C_5H_4NBr

C	5.58084300	-1.27690000	-0.35195400
C	4.19478500	-1.33246200	-0.53088800
C	3.41644200	-0.32810300	0.04782400
C	4.04998800	0.68147400	0.77522600
C	5.44323200	0.63593700	0.88887500
N	6.21462700	-0.31795600	0.34246100
H	6.21646900	-2.05102400	-0.79491900
H	3.73872600	-2.13832900	-1.10748800
H	3.47773000	1.48381900	1.24268900
H	5.96720900	1.41442000	1.45365000
Br	1.49560000	-0.33449600	-0.15234200
C	-1.57416300	-0.10176600	-0.41556600
C	-1.54609200	3.30786000	0.84350900
C	-1.04596500	3.47521300	-0.40949900
H	-1.69679700	4.02657200	1.64081000
H	-0.69511600	4.37131800	-0.90809500
C	-1.56491100	1.25125000	-0.18572400
N	-1.88434100	1.96227400	0.98477100
N	-1.05289700	2.23704900	-1.03776900
C	-2.22035100	1.30048600	2.22806300
H	-3.26421000	0.94833200	2.24346100
H	-1.56618100	0.42330400	2.35849600
H	-2.06955400	1.99968700	3.06055200
C	-0.64185700	1.96226000	-2.39804700
H	-0.48791200	0.87637600	-2.47425900
H	-1.41459900	2.26407500	-3.12521200
H	0.29550800	2.48912500	-2.62593500
C	-4.56442100	-2.08254900	0.17641400
C	-3.59005300	-3.02598200	0.23854700
H	-5.64059900	-2.17947900	0.26366500
H	-3.66117100	-4.09491800	0.40328100
C	-2.54234200	-1.00715300	-0.08273900
N	-3.94380700	-0.84062200	0.01670700
C	-4.61618000	0.36398300	-0.42069400
H	-4.43201700	1.20938800	0.25947600
H	-4.26158900	0.65595100	-1.42452200
H	-5.69647900	0.17430400	-0.46018800
C	-1.06944100	-3.02275200	0.01978900
H	-0.45085000	-2.78607400	0.89989400
H	-1.20953800	-4.10975400	-0.04439300
H	-0.53292300	-2.66014400	-0.87024000
N	-2.36489600	-2.38565800	0.09417700

$C(NHC_{Me})_2$-C_5H_4NI

C	5.65399000	-1.12943800	-0.40960300
C	4.26431600	-1.20012400	-0.55594900
C	3.47498200	-0.22039200	0.05401300
C	4.12090800	0.78347900	0.78217100
C	5.51739400	0.75982300	0.86470700
N	6.29121600	-0.17287400	0.28551500
H	6.29077900	-1.88712800	-0.87984100
H	3.81491000	-2.00755400	-1.13762400
H	3.55555900	1.57360800	1.28048500
H	6.04322000	1.53708100	1.43053600
I	1.31404600	-0.25719900	-0.12054600
C	-1.62188000	-0.10992900	-0.30035900
C	-2.08172100	3.30014800	0.87984600
C	-1.49468700	3.51182200	-0.32809100
H	-2.37866200	4.00276400	1.64984000
H	-1.19701900	4.43608300	-0.80935000
C	-1.77712100	1.25021700	-0.10620400
N	-2.26883300	1.92681300	1.01783400
N	-1.30959600	2.27507800	-0.93047300
C	-2.66659600	1.24414900	2.23261800
H	-3.69033400	0.84235600	2.16753200
H	-1.97960800	0.40143000	2.40631700
H	-2.61155700	1.94566600	3.07473500
C	-0.79513700	2.05400400	-2.26828200
H	-0.72243100	0.96760700	-2.41078000
H	-1.47375300	2.47907600	-3.02570100
H	0.20283000	2.50103200	-2.37975300
C	-4.59573700	-2.18916200	-0.01805700
C	-3.60999200	-3.09601900	0.19761400
H	-5.67120800	-2.31711900	-0.06046500
H	-3.66729500	-4.16129000	0.38836000
C	-2.58862600	-1.05793100	-0.06919900
N	-3.99400200	-0.93612600	-0.15620200
C	-4.65508700	0.23216600	-0.69807800
H	-4.66989300	1.07271700	0.01295200
H	-4.13446500	0.56834300	-1.61037500
H	-5.69017600	-0.03108700	-0.95087500
C	-1.09121700	-3.03476200	0.31251400
H	-0.52871500	-2.58902800	1.14584300
H	-1.22598400	-4.10830600	0.49656200
H	-0.49930200	-2.88985600	-0.60390600
N	-2.39508000	-2.42048400	0.18139800
